# Neocarzilin A induces apoptosis and mitochondrial disturbance by targeting reticulon 4-mediated endoplasmic reticulum stress

**DOI:** 10.1038/s41420-025-02560-3

**Published:** 2025-06-16

**Authors:** A. T. Jauch, J. Sailer, J. Braun, E. Czeslik, J. Geyer, C. Eberhagen, A. M. Vollmar, H. Zischka, S. A. Sieber, S. Zahler

**Affiliations:** 1 Department of Pharmacy, Pharmaceutical Biology, Ludwig-Maximilians-University of Munich, Munich, Germany; 2 Technical University of Munich, TUM School of Medicine and Health, Institute of Toxicology and Environmental Health, Munich, Germany; 3 Technical University of Munich, TUM School of Natural Sciences, Department of Bioscience, Center for Functional Protein Assemblies (CPA), Garching bei München, Germany; 4 Helmholtz Center Munich, Institute of Molecular Toxicology and Pharmacology, Neuherberg, Germany

**Keywords:** Energy metabolism, Target identification

## Abstract

Natural compounds are a valuable source of highly active biomolecules for the discovery of innovative drug targets as well as drug leads. The natural compound neocarzilin A (NCA) exhibits pronounced antiproliferative and antimigratory activity, which we previously ascribed to the target proteins vesicle amine transporter protein 1 (VAT-1) and bone marrow stromal antigen 2 (BST-2). We here additionally demonstrate the perturbation of mitochondrial functions (fragmentation of mitochondrial networks, ultrastructural changes, increased Opa1 splicing, loss of mitochondrial membrane potential, and excessive ROS generation) upon treatment with NCA. We observe impairment of the electron transfer chain and diminished ATP synthesis. Furthermore, NCA triggers apoptosis *via* activation of caspase-8, enhanced Bid processing, and cytochrome *c* release from mitochondria into the cytosol, leading to the activation of caspase-3 and -9 and, finally, PARP cleavage and DNA fragmentation. Endoplasmic reticulum (ER) stress is induced by treatment with NCA, and subsequently, the unfolded protein response (UPR) *via* the protein kinase r-like ER kinase (PERK) branch is prompted. Proteomic ABPP data indicate reticulon 4 (Rtn4, Nogo), an ER-located protein mainly involved in shaping ER tubules and maintaining proper ER function, as a promising hit to explain those effects. This novel molecular target was verified by co-staining of the target probe NC-4 and Rtn4, as well as RNA interference experiments, which resulted in reduced responsiveness of HeLa cells to NCA treatment. We propose NCA as a powerful tool to study the biology of Rtn4, and to develop more specific modulators of reticulons in the future. Furthermore, we introduce—to our knowledge—the first small molecular modulator of reticulon proteins.

## Introduction

Natural compounds provide a rich source of highly potent molecules to address biological targets [1], since they co-developed in physiological systems and, thus, exhibit evolutionary optimized properties for binding to their targets with high affinity and specificity [2]. However, there is still an urgent need for the exploration of suitable new drug targets, since only a minor set of human proteins (approx. 10%) is addressed by current therapeutics [3]. Here, too, natural substances show their strength, as they can serve as chemical tools for the elucidation of new druggable pathways [4]. For a long time, according to the one drug-one target paradigm, the standard strategy of medicinal chemists was to create highly potent and specific drugs. In recent decades, this has changed, and so-called multimodal drugs (also referred to as multi-target drugs) have attracted much attention as they bear the advantage of overcoming drug resistance and, therefore, offer new treatment options for diseases like chronic inflammation and cancer [5].

NCA, a short, chlorinated polyenone originating from the actinomycetes *Streptomyces carzinostaticus* [6], has been shown to exhibit potent antiproliferative [7] and antimigratory effects [8, 9] in cancer cells by addressing BST-2, a protein mainly known in the context of antiviral defense [10], and the largely uncharacterized VAT-1 firstly described as an integral membrane protein from *Torpedo californica* [11], respectively.

The endoplasmic reticulum (ER), among other functions, serves as a detoxification platform within the cells. On the one hand, perturbation of its proper function leads to cellular stress conditions, inducing response programs, and, if they fail to contract the stressors, eventually end up in cell death [12]. On the other hand, the ER is in close contact with mitochondria in so-called ER-mitochondrial contact sites (ERMCSs), which recently attracted much attention, and we are just starting to understand the highly dynamic nature of those foci [13] and their implementation in pathological conditions [14]. The affection of the ER can spread to mitochondria, thereby disturbing their functionality. This, in turn, can reinforce the induction of cell death pathways like intrinsic apoptosis resulting in a dual activation, which might be exploited pharmacologically [15] to bypass one of the hallmarks of cancer cells, the ability to evade apoptosis [16].

Reticulons are a family of proteins located in ER membranes [17] where they are mainly involved in shaping ER tubes [18]. The mammalian reticulon family comprises four members (Rtn1, 2, 3, and 4), which exhibit a highly conserved C-terminal domain called reticulon homology domain (RHD) [19]. However, the amino-terminal region differs greatly, most probably accounting for the various functions they have besides stabilizing high-curvature regions of the ER.

Here we describe the actions of NCA on ER- and mitochondrial function, which cannot be ascribed to previously identified targets of NCA. We propose Rtn4 as an additional target of NCA and suggest NCA as an interesting natural product to cause cell death in an unconventional way.

## Results

### Neocarzilin A affects network organization and ultrastructure of mitochondria

**Fig. 1:**
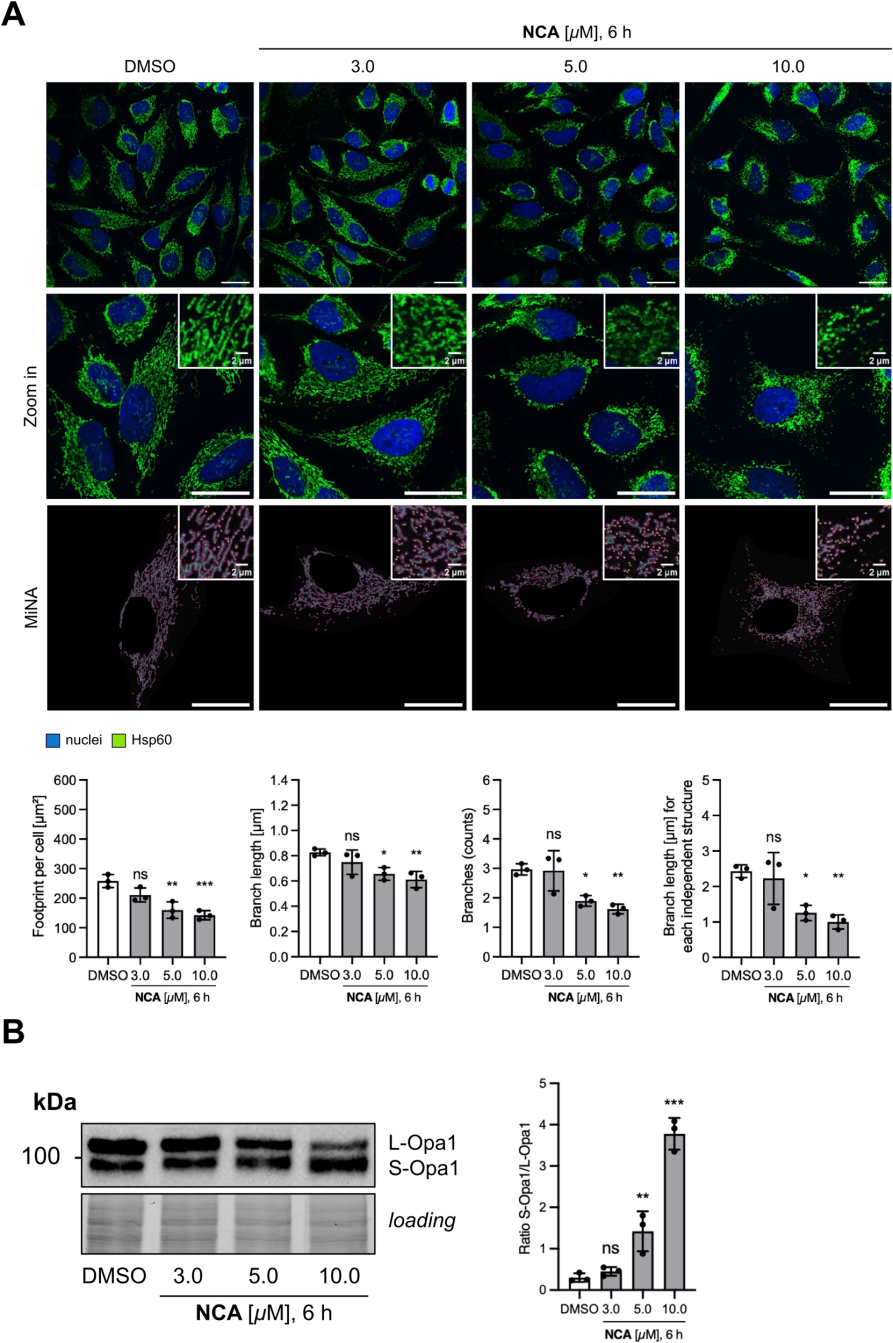
Effect of NCA on mitochondrial dynamics. **A** Mitochondrial networks were analyzed by immunostainings after treatment with NCA or DMSO for 6 h at the indicated concentrations. Representative images on top panel, nuclei shown in blue, Hsp60 in green (scale bar 25 µm). Brightness was adjusted to improve visibility. Bottom row shows MiNA analysis, purple area represents mitochondrial footprint, green lines mitochondrial length, blue dots connection sites, and yellow dots the end of network structures. **B** Protein expression of optic atrophy protein 1 (OPA1) assessed by Western blotting. Left panel shows representative blot. Normalized data (**A**) and ratio of S- over L-splicing form **B** are presented in bar graphs as mean ± SD, *n* = 3. Statistical significance was analyzed by one-way ANOVA with Dunnett’s posttest compared to mean of DMSO control (ns not significant, *P < 0.05, **P < 0.01, ***P < 0.001).

First, we analyzed the mitochondrial network in HeLa cells after treating the cells for 3 h and 6 h with NCA compared to DMSO control cells (Fig. 1A and S1) by immunostaining Hsp60. Total mitochondrial footprint, mean branch length, branch count, and summed branch length as characteristics describing the network decreased in a time- and dose-dependent manner, significantly after 6 h stimulation. This reflects a breakdown of the mitochondrial network. Similar results were obtained using MitoTracker^™^ Deep Red FM (Fig. S2), however, with this approach, evaluation with the MiNA tool failed. An indirect effect on mitochondrial morphology *via* the cytoskeleton could be ruled out by immunostaining of the microtubules, showing no alteration upon NCA treatment (Fig. S3), which implied that the compound directly targets the process of mitochondrial fusion and fission. To elucidate the underlying mechanisms, immunoblotting of known regulators of mitochondrial fission and fusion was performed. Surprisingly, neither mitofusin 1 (Mfn1) responsible for the fusion of the outer mitochondrial membrane, nor dynamin-related protein 1 (Drp1), which facilitates fission processes, changed in protein levels upon treatment with the compound (Figs. S4 and S5).Of note, the ratio of the short over long isoform of optic atrophy protein 1 (OPA1), the main regulator of the inner mitochondrial membrane fusion, increased upon treatment (Fig. 1B), indicating enhanced splicing activity, and most probably accounting for the observed fragmented phenotype. However, detailed analysis of the localization or activity of OPA1 would be needed to address this point more clearly.

**Fig. 2:**
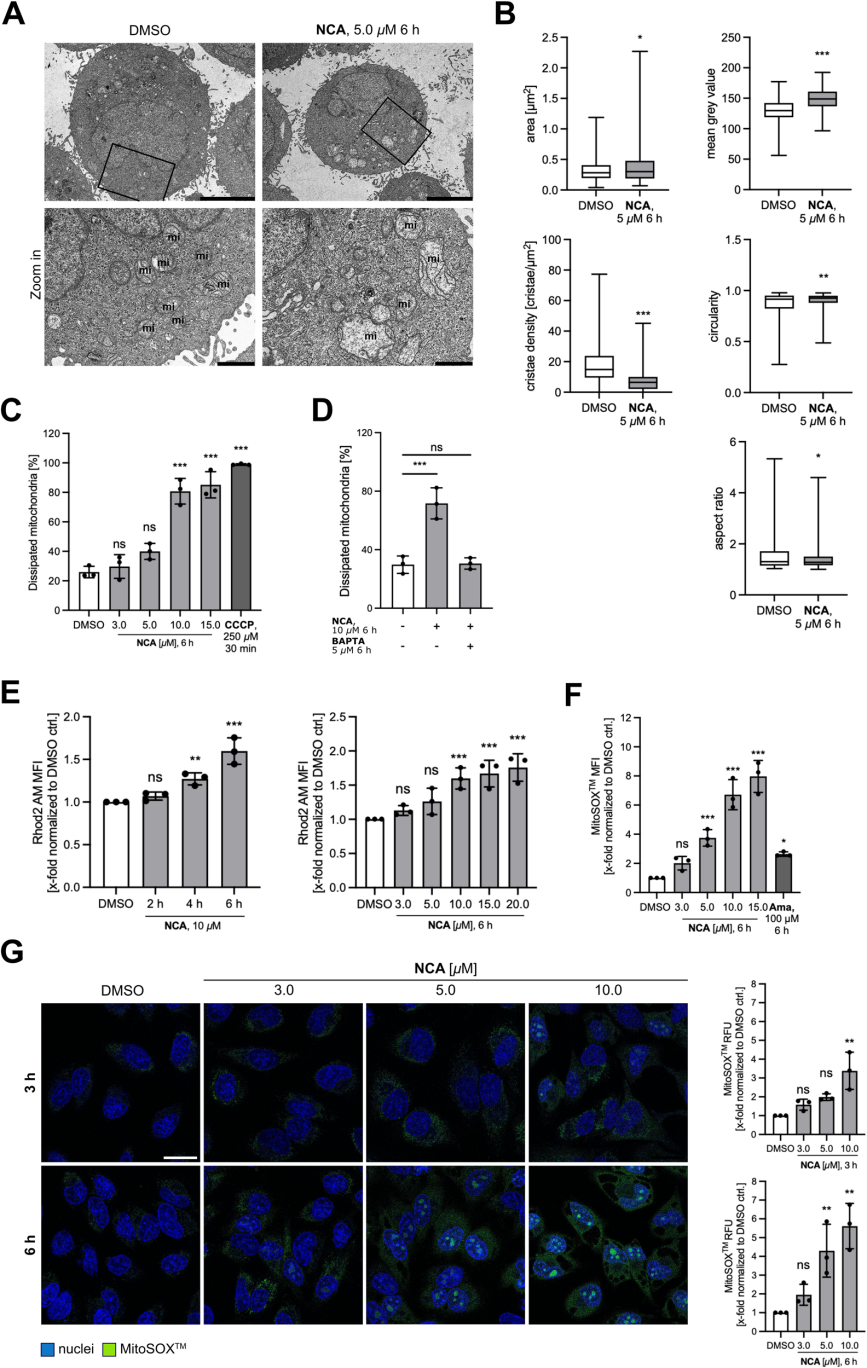
NCA changes mitochondrial ultrastructure, disturbs mitochondrial membrane potential and increases ROS generation. **A** Representative transmission electron micrographs of HeLa cells treated with NCA or DMSO as indicated (scale bar top row 5000 nm, bottom row 1000 nm, mi=mitochondria). **B** Quantification of 50 mitochondria per repetition of mitochondrial size, mean gray value, cristae density and shape factors circularity and aspect ratio. **C**, **D** Analysis of mitochondrial membrane potential via JC-1 staining and flow cytometry upon **C**, **D** NCA or DMSO treatment and **D** concomitant BAPTA application. CCCP served as positive control. **E** Mitochondrial calcium levels upon treatment with NCA or DMSO as indicated by using Rhod2-AM dye and flow cytometry. **F**, **G** Mitochondrial superoxide generation after NCA or DMSO treatment as indicated measured with the MitoSOX^TM^ dye by **F** flow cytometry and **G** live cell imaging. **F** Antimycin A served as positive control. Representative images shown on the bottom panel, nuclei in blue, MitoSOX in green (scale bar 25 *µ*m). Brightness was adjusted to improve visibility. Absolute values **B**–**D** or normalized data **E**–**G** are presented in **B** box and whiskers (min to max) or **C**–**G** bar graphs as mean ± SD, *n* = 3. Statistical significance was analyzed by **B** two-tailed unpaired Student’s *t*-test or **C**–**G** one-way ANOVA with Dunnett’s posttest compared to mean of DMSO control (ns not significant, *P < 0.05, **P < 0.01, ***P < 0.001).

Next, we assessed the ultrastructure of NCA-treated HeLa cells by transmission electron microscopy (TEM) and compared them to DMSO control cells. It turned out that the natural compound induced enlargement of mitochondria, accompanied by reduced cristae structure, lower matrix density, and a rounder morphology (Fig. 2A, B)

### Mitochondrial membrane potential is dissipated and ROS generation elevated by NCA

NCA caused a prominent dissipation of the mitochondrial membrane potential Δψm, comparable to the positive control CCCP in the highest applied concentrations (Fig. 2C). Concomitant administration of the calcium chelator BAPTA could completely rescue this effect (Fig. 2D), which led to the hypothesis that the mitochondrial effects of the compound are driven by mitochondrial calcium overload. We supported this idea by measuring mitochondrial calcium levels. This revealed a time- and dose-dependent increase in mitochondrial calcium (Fig. 2E) strongly emphasizing the pivotal contribution of calcium to this phenotype.

Perturbation of the Δψm is often accompanied by changes in mitochondrial ROS formation [20], which we then examined with the use of the MitoSOX dye in flow cytometry and an imaging-based assay (Fig. 2F, G). In the former, the maximum applied NCA dose provoked an increase of mitochondrial superoxide that even surpassed the effect of the positive control antimycin A (Ama). This finding was visually supported by the live cell imaging, which showed elevated mROS levels already after treatment for 3 h.

### Neocarzilin A disturbs respiratory chain function leading to reduced ATP production

**Fig. 3:**
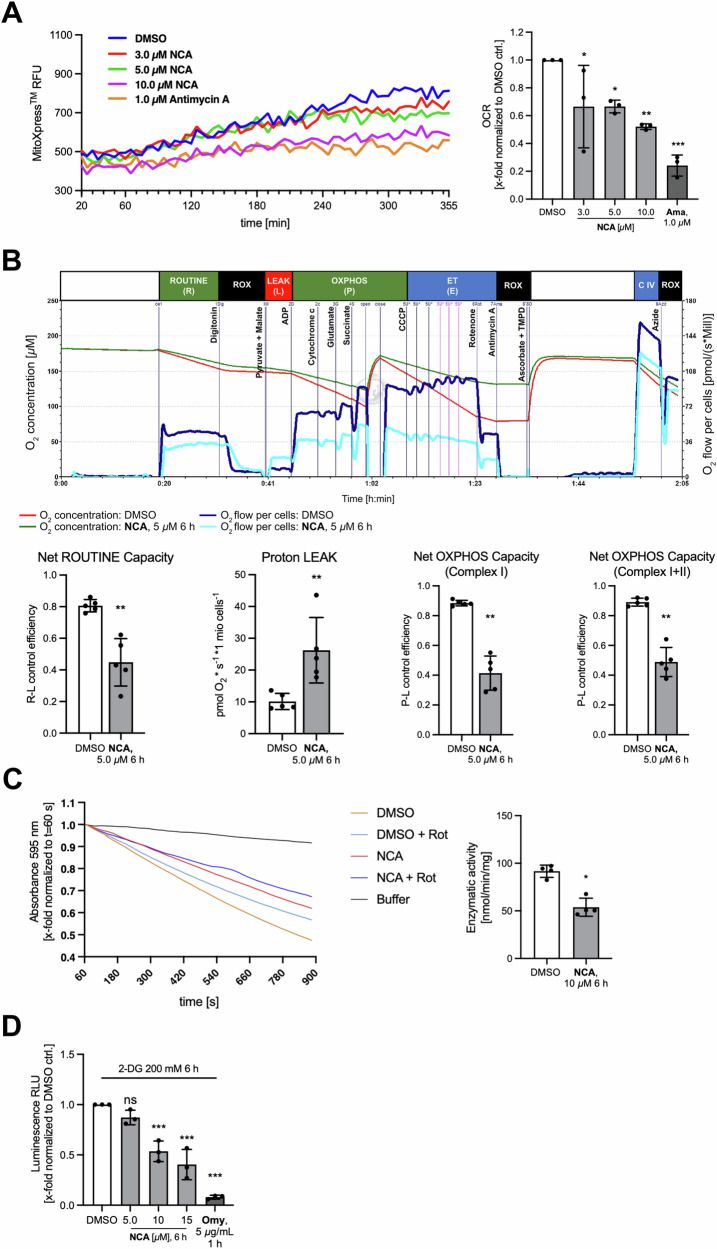
Electron transfer chain perturbation by NCA. **A** Oxygen consumption rate (OCR) of HeLa cells treated with NCA, DMSO, and antimycin A as positive control was assessed in a plate reader assay over 6 h at 37 °C using an oxygen-quenchable fluorescent dye. OCR was calculated from the slope of the recorded curves. **B** High-resolution respirometry measurements of 6 h NCA or DMSO-treated cells following SUIT protocol 008_O2_ce-pce_D025 in an Oroboros O2k instrument. **C** Complex I activity of mitochondria enriched from HeLa cells treated with NCA or DMSO as indicated was measured by NADH-dependent indirect reduction of DCIP using decylubiquinone as substrate. Decrease in absorbance at 595 nm was recorded over 15 min in a plate reader, not complex I-related conversion was assessed by applying rotenone. Enzymatic activity was calculated from the slope of the linear section of the curves and the protein amount. **D** CellTiter-Glo assay to determine ATP content of NCA or DMSO stimulated HeLa cells as indicated. Glycolysis was inhibited by concomitant 2-deoxyglucose (200 mM, 6 h) treatment. **C** Absolute values, **A**, **D** normalized data and **C** control efficiencies are presented in bar graphs as mean ± SD, **A**, **D** *n* = 3, **C** *n* = 4, **B** *n* = 5. Statistical significance was analyzed by **B**, **C** two-tailed unpaired Student’s t-test or Mann–Whitney test or **A**, **D** one-way ANOVA with Dunnett’s posttest compared to mean of DMSO control (*P < 0.05, **P < 0.01, ***P < 0.001).

Oxygen consumption rate (OCR) was reduced after stimulation with NCA (Fig. 3A). To dig deeper into the characterization of the respiratory chain, high-respirometry was performed. The applied protocol allowed the assessment of the ROUTINE and LEAK respiration, as well as complex I and combined complex I and II activity (Figs. 3B and S6). Stimulation with NCA for 6 h triggered a mild reduction of the ROUTINE, but a very prominent increase in the LEAK and reduced CI and CI + II activity. Moreover, we were able to induce this effect directly by titrating higher doses of the compound (>50 µM) into the measuring chamber (Fig. S7). To substantiate those findings, we measured NADH:ubiquinone-dehydrogenase (complex I) enzymatic activity in enriched mitochondria fractions from NCA-treated HeLa cells (Fig. 3C). This in vitro enzymatic assay resulted in a significantly reduced activity of complex I by almost 50%. Eventually, an intriguing impairment of ATP production, was shown in a luminescence assay (Fig. 3D). However, this was only detectable after manipulation with the glycolysis inhibitor 2-DG since HeLa cells seem to meet their ATP demand rather by anaerobic glycolysis than by OXPHOS, in line with Otto Warburg’s hypothesis (Fig. S8) [21]. At the same time, a cytotoxic effect under those treatment conditions that would affect the ATP readout could be ruled out by crystal violet staining (Fig. S9).

### Endoplasmic reticulum stress is triggered by neocarzilin treatment

**Fig. 4:**
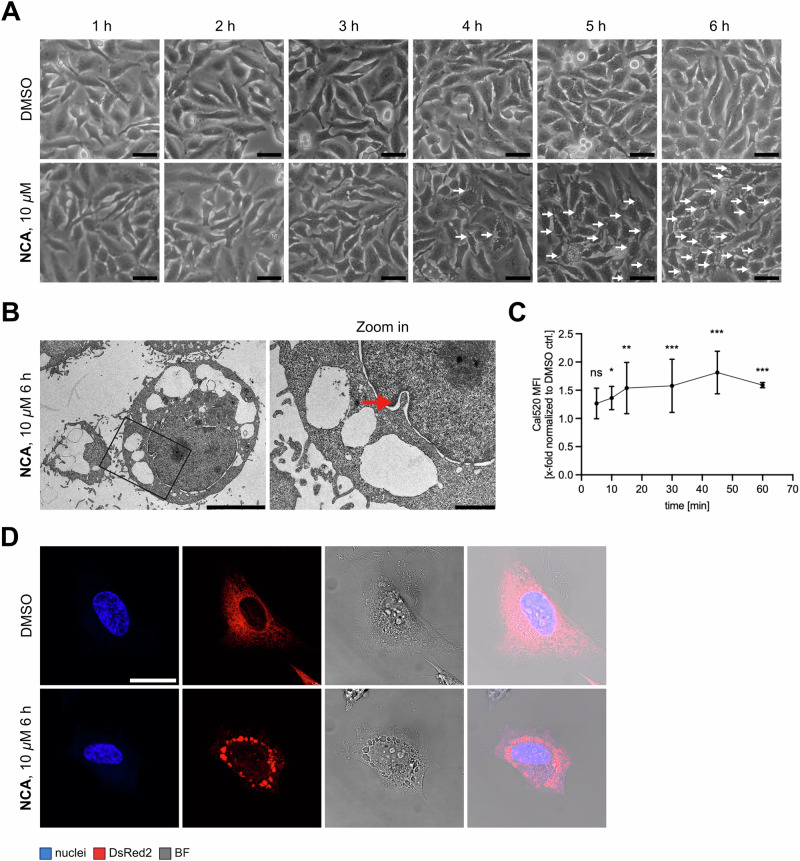
NCA treatment leads to ER-derived cytosolic vacuolization and calcium increase. **A** Representative phase contrast images of HeLa cells treated with NCA or DMSO as indicated (scale bar 25 µm). White arrows indicate vacuoles. **B** Representative transmission electron micrographs of HeLa cells treated with NCA as indicated (scale bar left image 5000 nm, right image 1000 nm). Red arrow indicates nuclear membrane budding. **C** Analysis of cytosolic calcium by Cal520 AM flow cytometry assay. HeLa cells were loaded with the dye and treated with 10 µM NCA for the indicated times. Cal520 AM mean fluorescence was recorded. **D** Representative confocal images of HeLa cells transfected with DsRed2 plasmid, treated with 10 µM NCA for 6 h and analyzed by live cell imaging. Nuclei shown in blue, ER in red, bright-field (BF) shown to display coincidence of ER dye and vacuoles (scale bar 25 µm). **C** Data are presented in an XY graph as mean ± SD, *n* = 3. Statistical significance was analyzed by two-way ANOVA with Tukey’s posttest compared to mean of the corresponding DMSO control (ns not significant, *P < 0.05, **P < 0.01, ***P < 0.001).

In addition to the morphological changes of the mitochondria, the compound also induced a pronounced cytoplasmic vacuolization observable by phase contrast microscopy (Fig. 4A). This became even more evident in electron micrographs recorded under the same treatment condition (Fig. 4B). At the same time, the vacuoles could clearly be identified to bud off the nuclear envelope (red arrow), making it very likely that they are endoplasmic reticulum derived. To prove this, the fluorescent protein DsRed2 modulated with an ER signaling peptide and retention motif to ensure its sole ER expression was expressed in HeLa cells before applying the natural compound (Fig. 4D). Live cell imaging showed a concurrence of the induced vacuoles and the dye, confirming the hypothesis that they originated from the ER. To exclude autophagy involvement, common markers were assessed by immunoblotting. However, these showed no significant changes upon NCA treatment (Fig. S10). We speculated that this intriguing dilatation might lead to an increase in cytosolic calcium since it could be accompanied by changes in membrane permeability of the ER. Indeed, significantly elevated cytosolic calcium levels could be demonstrated upon treatment with the natural compound (Fig. 4C), which could provide a source for the observed increase in mitochondrial calcium and a possible explanation for the described mitochondrial effects of NCA.

**Fig. 5:**
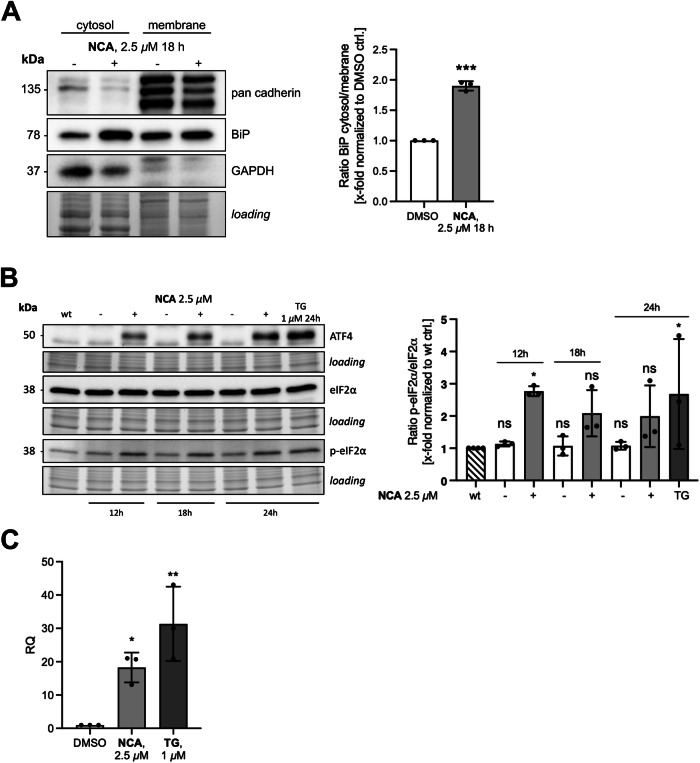
ER stress is triggered upon NCA stimulation. **A** Subcellular fractionation of HeLa cells treated with NCA or DMSO as indicated with subsequent immunoblotting for ER chaperone BiP. **B** ER stress markers (eIF2α + ATF4) of HeLa cells treated with NCA or DMSO as indicated, Thapsigargin (TG) served as positive control. **C** CHOP mRNA levels of HeLa cells treated with NCA or DMSO as indicated for 18 h analyzed by qPCR, TG served as positive control. Relative quantification via ΔΔCt method. **A**–**C** Data are presented in bar graphs as mean ± SD, *n* = 3. Statistical significance was analyzed by **A** two-tailed unpaired Student’s *t*-test or **B**, **C** one-way ANOVA with Dunnett’s posttest compared to mean of **B** wt group or **C** DMSO control (ns not significant, *P < 0.05, **P < 0.01, ***P < 0.001).

Since ER dilatation is a common phenomenon observed during ER stress, we next focused on markers of this event upon compound treatment. The localization of the ER chaperon binding immunoglobulin protein (BiP) was investigated by subfractionation (Fig. 5A). A significant re-distribution from the 100,000 × g pellet to the corresponding supernatant, assuming a trans-localization from the ER-containing fraction to the cytosol, could be observed, which was previously described as result of elevated ER membrane permeability and loss of ER integrity during ER stress [22, 23]. Next, the markers eukaryotic initiation factor 2α (eIF2α) and activating transcription factor 4 (ATF4) were assessed by immunoblotting (Fig. 5B), which demonstrated a strong activation of the protein kinase r-like ER kinase (PERK) pathway of the unfolded protein response (UPR), eventually leading to the upregulation of the transcription factor CHOP on mRNA level, which could be shown by qPCR (Fig. 5C).

### Unresolved ER stress and prolonged mitochondrial disturbance lead to induction of extrinsic apoptosis

**Fig. 6:**
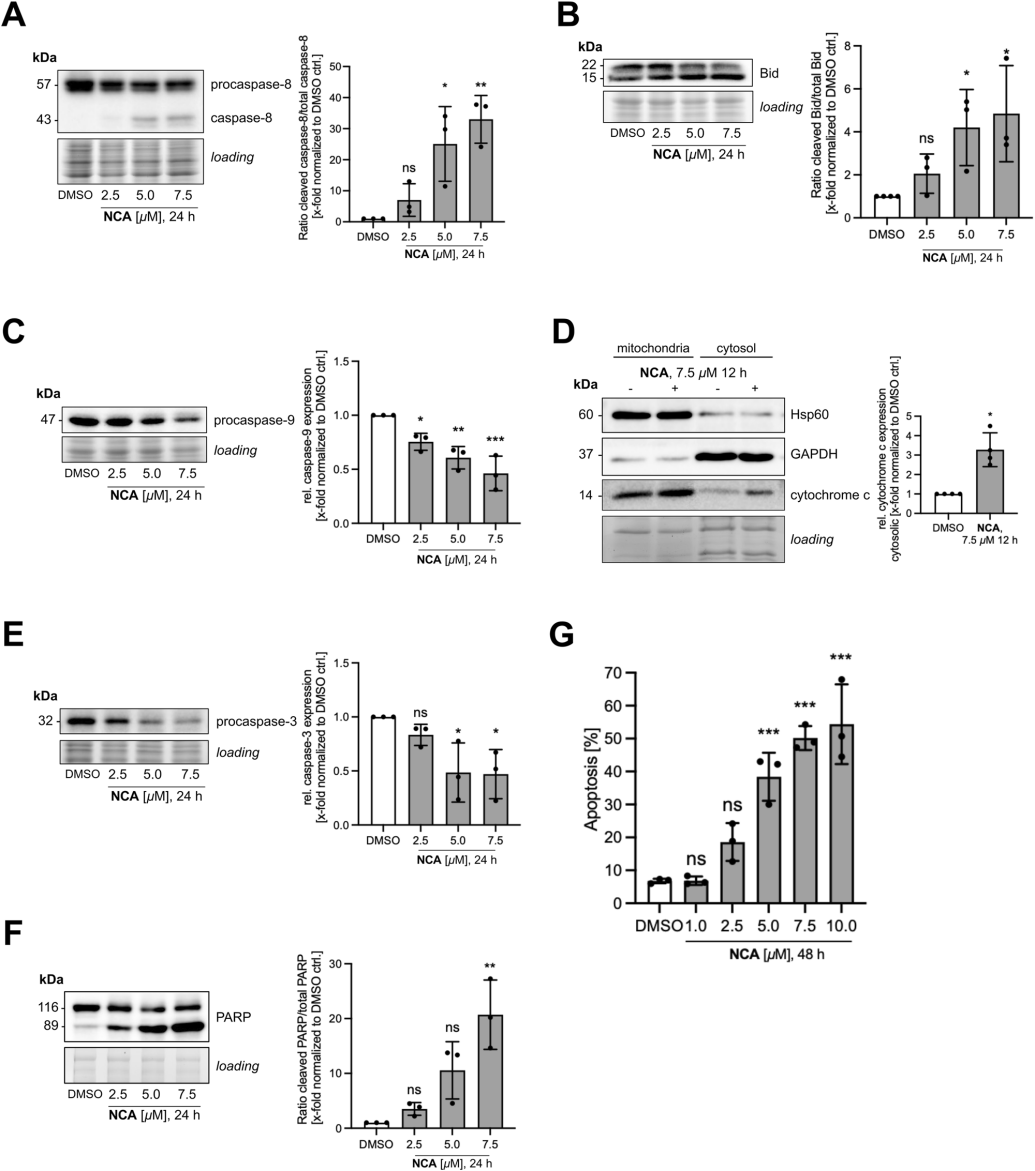
Induction of intrinsic apoptosis pathways by NCA. **A**–**F** Analysis of apoptosis markers by immunoblotting. HeLa cells were treated with NCA or DMSO as indicated and ratio of cleaved to total **A** caspase 8, **B** Bid and **F** PARP or relative expression of **C** caspase 9, **E** caspase 3 were determined. **D** Cytochrome c release was analyzed by subcellular fractionation and immunoblotting in mitochondrial and cytosolic fractions. **G** DNA fragmentation was analyzed by propidium iodide staining and flow cytometry. **A**-**G** Data are presented in bar graphs as mean ± SD, *n* = 3. Statistical significance was analyzed by **A**, **B**, **C**, **E**, **F**, **G** one-way ANOVA with Dunnett’s posttest or **D** Mann-Whitney test (ns=not significant, *P < 0.05, **P < 0.01, ***P < 0.001).

On the one hand, mitochondria play a crucial role in orchestrating the programmed cell death apoptosis. On the other hand, ER stress is known to induce apoptosis if protein homeostasis cannot be restored, and cellular stress exceeds a certain threshold. The so far observed effects of NCA on both organelles suggested that the compound could trigger programmed cell death, so we analyzed common apoptosis markers by Western blotting. NCA causes an increased splicing of Bid (Fig. 6B) by caspase-8 (Fig. 6A), which led to a translocation of cytochrome c from the 10,000 × *g* pellet to the 100,000 × *g* supernatant (Fig. 6D), indicating its release from mitochondria to the cytosol, assessed by subcellular fractionation. This activated caspase-9 (Fig. 6C) and executioner caspase-3 (Fig. 6E), induced PARP cleavage (Fig. 6F), and finally led to DNA fragmentation, demonstrated in a PI Nicoletti assay (Fig. 6G).

### Proteomic ABPP revealed reticulon 4 protein as a possible target

The so far identified targets of NCA, VAT-1 and BST-2 have previously been related to mitochondria, ER stress, ER-mitochondria communication and apoptosis [24–27]. It was, therefore, reasonable to assume that they could, at least in part, also be involved in this novel phenotype. However, we ruled this out by applying the natural compound in the respective knockout models, which resulted in elevated ROS generation comparable to the effect in HeLa wt cells (Fig. S11). Also, the fragmented network morphology could be provoked by NCA in BST-2 KD cells (Fig. S12) and VAT-1 KO cells (Fig. S13). Furthermore, NCA treatment led to cytoplasmic vacuolization in VAT-1 lacking cells, comparable to HeLa wt (Fig. S14).

**Fig. 7:**
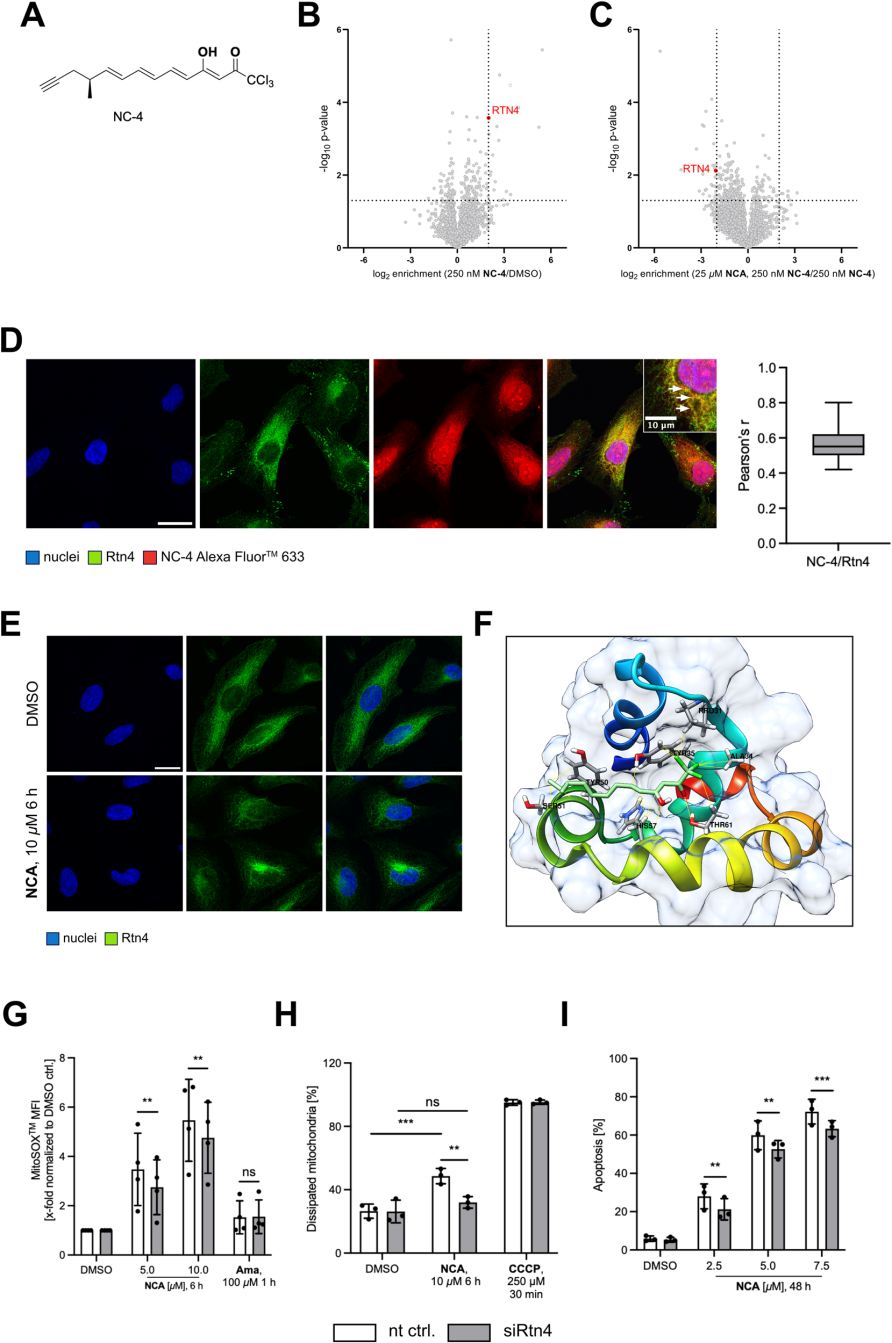
Target identification and validation disclose reticulon 4 as responsible molecular target. **A** Chemical structure of target probe NC-4, drawn with ChemDraw 20.0 **B** Target identification by ABPP with NC-4 target probe in HeLa cells, **C** respective competition experiment. **D**, **E** Representative images of immunofluorescence staining of HeLa cells **D** incubated with NC-4 and clicked to Alexa Fluor^TM^ 647 azide or **E** treated with NCA or DMSO as indicated and **D**, **E** immunostained for Rtn4. **D**, **E** Nuclei shown in blue, Rtn 4 in green, **E** Alexa Fluor^TM^ 647 coupled NC-4 in red (**D**, **E** scale bar 25 µm), *n* = 3. **F** Molecular docking of nogo-66 and NCA by UCSF Chimera and AutoDock Vina plugin. Contact sites are marked yellow, hydrogen bond is marked red, with interacting residues labeled. Flow cytometry experiments in Rtn4 knockdown HeLa cells to determine ROS generation (**G**), mitochondrial membrane potential (**H**), and apoptosis induction (**I**) upon NCA or DMSO treatment as indicated. Data are presented A,B in volcano plots, C box and whiskers (min to max), **F**–**H** bar graphs as mean ± SD, **B**, **C**, **G** *n* = 4, **D**, **H**, **I** *n* = 3. Statistical significance was analyzed by two-way ANOVA with Tukey’s posttest (ns not significant, **P < 0.01, ***P < 0.001).

In search of a fitting target candidate, we re-analyzed activity-based protein profiling (ABPP) data from a previous study [7]. In this study, the probe NC-4 (Fig. 7A) was used to identify potential target proteins. It exhibits a highly similar structure to the mother compound except for the alkine handle, which can be used for conjugation to biotin and subsequent avidin enrichment. The data (PRIDE database number: PXD050453**)** propose an interesting target candidate, reticulon 4 (Rtn4), also known as neurite outgrowth inhibitor (Nogo), which was highly enriched (Fig. 7B). In competition experiments from the same study (Fig. 7C), where cells were pre-incubated with the original compound NCA in a 100-fold excess compared to the target probe, Rtn4 was significantly depleted, emphasizing the validity of the hit. Importantly, the target probe was proven to be able to induce the vacuolization in HeLa cells (Fig. S15), although higher concentrations were required. This reflects the decrease in potency, which is frequently experienced when modifying natural compounds for probe-based target ID approaches. For further verification, cellular NC-4 distribution was visualized by clicking it on an Alexa Fluor 647 azide, followed by immunostaining for Rtn4 (Fig. 7D). Co-localization analysis resulted in a mean Pearson’s r value of 0.56, indicating a strong correlation of the signals and again underpinning Rtn4 as an additional molecular target of NCA. At the same time, treatment of HeLa cells with the natural compound obviously changed the distribution of Rtn4 expression shown in an immunofluorescence staining (Fig. 7E), which corroborates the interaction of NCA and Rtn4. The structure of the cytoplasmic domain of Nogo, Nogo-66, and NCA were then in silico docked using UCSF Chimera and Autodock Vina (Fig. 7F). Here, a docking score of −5.5, one hydrogen bond between the carbonyl oxygen atom of NCA and Thr 61 (red line), and several hydrophobic interactions (yellow lines) were found, further supporting the hypothesis that NCA addresses Rtn4. Next, RNA interference was used to downregulate Rtn4 protein expression, and subsequently, mitochondrial superoxide generation, mitochondrial membrane dissipation, and apoptosis induction upon NCA treatment were again assessed by flow cytometry (Fig. 7G–I). In all cases, the responsiveness of Rtn4 knockdown cells was significantly reduced compared to non-targeting control cells.

## Discussion

ER-mitochondria contact sites are well-established exchange platforms that allow the transfer of biological building blocks and, most importantly, calcium [28], which is required to drive the conversion of ADP + P_i_ to the cellular energy currency ATP [29]. Recently, these foci attracted much attention, bearing an addressable site for therapeutic intervention, since these contact sites are involved in various pathological conditions.

**Fig. 8:**
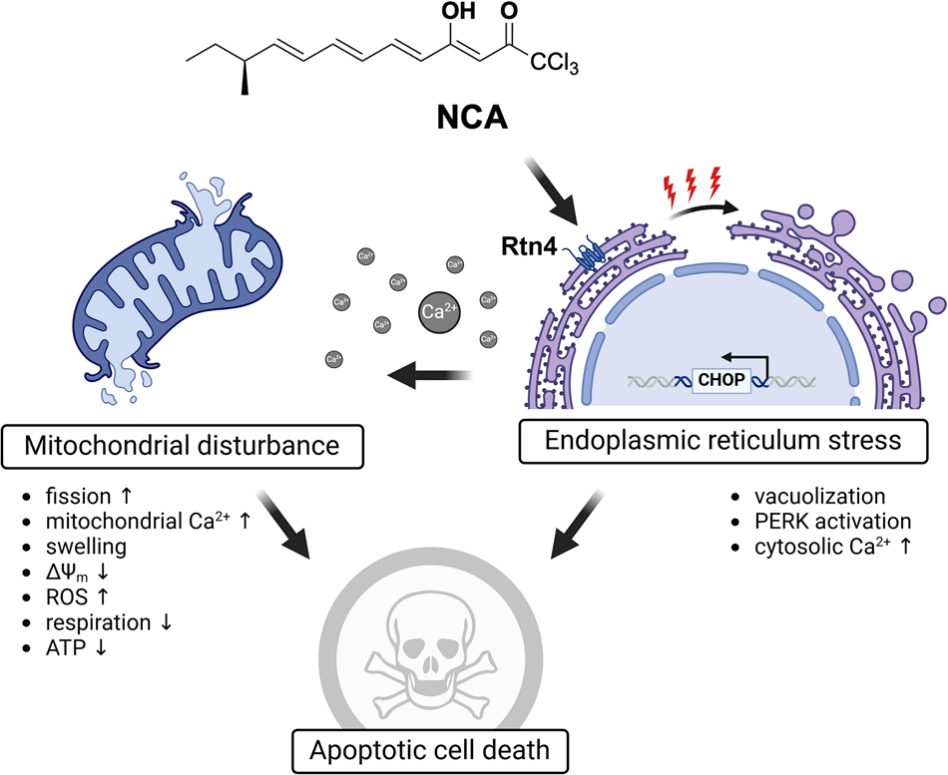
Proposed mechanism of action.

In the present study, we identified neocarzilin A as a novel small-molecule modulator of reticulon 4, which targets the interface of the ER and mitochondria by provoking endoplasmic reticulum stress and finally compromising mitochondrial functions, which ultimately leads to the induction of apoptotic cell death (Fig. 8).

This effect seems to be strongly driven by an unbalanced cellular calcium homeostasis, originating from the stressed ER, which leaps to mitochondria, thereby impairing vital functions in such a manner that the cancer cells eventually undergo programmed cell death.

However, it is also conceivable that, in addition to the newly identified Rtn4, there exist further targets, which contribute to the observed effects. This could also explain why RNA interference did not completely block sensitivity of the cells toward NCA. A further argument in favor of this assumption is that the proton LEAK could be provoked within a few minutes by administering high doses of the compound. This could indicate that a potential additional target could be directly localized in mitochondria. On the other hand, the increase in cytosolic calcium happened in this short time under the same treatment conditions, too (data not shown). Another explanation for the incomplete blockage of NCA action after silencing gene expression might be that NCA addresses not only Rtn4 but also other reticulon family members since they show high structural similarity, especially in the reticulon homology domain. This hypothesis is further substantiated by ABPP experiments with NC-4, which frequently hit additional reticulon proteins (e.g., Rtn1, Rtn3, Tables S1, S2 derived from previous studies [7]). In addition, only a knockdown efficiency of approx. 60% could be achieved (Fig. S16).

Previously, reticulon 4 has been shown to be considerably involved in the regulation of mitochondrial functions as well as cell death induction. However, its role in those processes remains controversial, as, e.g., Carter et al. reported increased mitochondrial hyperfusion upon silencing of Rtn4 [30]. Since in case of Rtn4 manipulation by NCA, we observed the opposite phenomenon (i.e., mitochondrial network fragmentation), we speculate that the compound rather modifies the protein’s function than inhibiting it. By binding to the cytoplasmic loop of Rtn4, NCA could change the conformation of the protein, altering its membrane topology and finally affecting its ER shaping function. The distance between ER and mitochondria could, thus, change, which has recently been demonstrated to be a decisive factor for the Ca^2+^ transfer [31]. However, the exact temporal sequence of calcium flux would have to be examined in more detail to substantiate the hypothesis that ER calcium is shuffled to mitochondria and mediates the observed phenotype.

It has also been reported that ER stress leads to a perinuclear redistribution of mitochondria, most probably via the microtubular network, thereby increasing the organelle’s coupling [32]. However, in our hands, microtubule involvement could be ruled out.

Already in 2008, Nogo-A was shown to interact with mitochondrial proteins influencing complex III activity [33]. Thus, besides the effect on mitochondria mediated through ER stress, a direct effect of our compound on mitochondrial functions by targeting Rtn4 is also conceivable.

Moreover, several publications have shown controlling functions of reticulon family members on Bcl-2 proteins, which could account for its implementation in cell death regulation. Nogo-B has been reported on the one side to negatively regulate extrinsic apoptosis and, on the other, to be a proapoptotic protein itself [34, 35]. Reticulon 1-C (Rtn1-C) is supposed to be involved in cerebral ischemia/reperfusion injury by provoking ER stress and activating mitochondrial apoptosis pathways [36]. All this emphasizes the strong link between this protein family, the ER, mitochondria, and programmed cell death.

In summary, we highlight NCA as a promising tool compound and elucidate its molecular target reticulon 4. Further investigation into the biological implementation of reticulon proteins in mitochondrial functions and cell death will be needed to exploit the potential of this novel, druggable target. Furthermore, additional targets of NCA should be explored to fully understand the nature of its mitochondrial toxicology.

## Materials and methods

### Cell lines and culture

HeLa cells were obtained from DSMZ (Braunschweig, Germany) and were cultured in Dulbecco’s Modified Eagle’s Medium high glucose (DMEM) (PAN Biotech, Aidenbach, Germany) supplemented with 10% (v/v) fetal calf serum (PAN Biotech) and grown at 37 °C with 5% CO_2_ in a humidified incubator (Heraeus, Hanau, Germany). Cells were passaged twice to thrice a week. Short tandem repeat analysis and mycoplasm testing were performed on a regular basis.

### Compounds

2-Deoxy-D-glucose (2 M in water) was purchased from Carl Roth GmbH (Karlsruhe, Germany). Antimycin A (10 mM in DMSO) and rotenone (2 µM in EtOH) were purchased from Enzo Life Science GmbH (Lörrach, Germany). Carbonyl cyanide m-chlorophenyl hydrazone (CCCP) (10 mM in DMSO) and probenecid (100 mM in DMSO) were purchased from Sigma-Aldrich (Taufkirchen, Germany). Oligomycin A (5 mg/mL in DMSO) was purchased from Merck Millipore kGaA (Darmstadt, Germany), and thapsigargin (10 mM in DMSO) was purchased from Santa Cruz Biotechnology (Dallas, TX, USA).

Neocarzilin A and its derivatives were synthesized as previously described [7].

### Confocal microscopy

For confocal imaging, cells were seeded into 8-well µ-slides (ibidi GmbH, Munich, Germany) and treated as indicated. For fixation, 4% PFA was applied, cells were permeabilized with 0.1% Triton-X-100, blocked with 2% BSA before staining with heat shock protein 60 (Hsp60) (1:200, sc-1052, Santa Cruz Biotechnology) and Rtn4 (1:200, sc-271878, Santa Cruz Biotechnology) primary antibodies and indirect immunofluorescence via an Alexa Fluor^TM^ 488 labeled secondary antibody (1:400, A-11001, Invitrogen). Nuclei were counterstained with Hoechst 33342 (100 µg/mL), then specimens were mounted with FluorSave reagent (Merck Millipore, Darmstadt, Germany) and sealed with a glass cover slip. Imaging was performed with a Leica TCS SP8 confocal laser scanning microscope (Leica Microsystems GmbH, Wetzlar, Germany) and LAS X software (Leica).

NC-4 probe localization was analyzed after incubation for 1 h, followed by conjugation to an AlexaFluor^TM^ 647 azide (10 µM, A-10277, Invitrogen) *via* click-chemistry (click mix: CuSO_4_ 1 mM, TBTA 100 µM, TCEP 1 mM in PBS) and co-staining with Rtn4 primary antibody (1:200, sc-271878, Santa Cruz Biotechnology) and the respective secondary Alexa Fluor^TM^ 488 labeled secondary antibody (1:400, A-11001, Invitrogen), nuclei were counter-stained with Hoechst 33342 (100 µg/mL). Co-localization was analyzed by the Coloc2 plugin in ImageJ and presented as Pearson’s *r* value.

Mitochondrial networks of treated and Hsp60-stained HeLa cells were analyzed using the MiNA ImageJ plugin as previously described [37].

For investigation of ER vacuoles, DsRed2-ER-5 plasmid (DsRed2-ER-5 was a gift from Michael Davidson, Addgene plasmid # 55836; http://n2t.net/addgene:55836; RRID:Addgene_55836) was transfected and incubated for 42 h before treating the cells with 10 µM NCA or DMSO for additional 6 h. After that, live cell imaging was performed.

Mitochondrial superoxide generation was assessed by loading the MitoSOX^TM^ Red dye (5 µM in HHBS, M36005, Invitrogen) after treating HeLa cells for 3 and 6 h with NCA or DMOS at the indicated concentrations.

### Flow cytometry

Mitochondrial membrane potential (Δψm) was measured using the lipophilic, cationic dye 5,5,6,6’-tetrachloro-1,1’,3,3’ tetraethylbenzimi-dazoylcarbocyanine iodide (JC-1). Cells containing mitochondria with intact ΔΨm exhibit red fluorescent (PE channel), and those with dissipated Δψm a green signal (FITC channel). Gating was set using DMSO as negative and carbonyl cyanide m-chlorophenylhydrazone (CCCP, 250 µM, 30 min) as positive control. Fluorophore spill-over was compensated.

For assessment of mitochondrial calcium, the Rhod2- acetoxymethyl (AM) ester dye (R1244, Invitrogen, 5 µM in HEPES buffered Hank’s buffer (HHBS), 1 mM probenecid, 0.02% Pluronic) was utilized according to the manufacturer’s instructions. Briefly, cells were loaded for 30 min at 37 °C protected from light prior to stimulation with NCA or DMSO as indicated.

Cytosolic calcium levels were determined with the Cal520-AM dye (21130, ATT Bioquest). For this, the calcium dye was loaded (5 µM in HHBS, 1 mM probenecid, 0.02% Pluronic) for 90 min at 37 °C protected from light, cells were collected, washed, and transferred to FACS tubes and treated with NCA or DMSO as indicated.

Apoptotic cell death of HeLa cells treated with NCA or DMSO for 48 h was investigated by propidium iodide (PI) staining, according to Nicoletti et al. [38]. Cells were collected, pelleted, and resuspended in hypotonic fluorochrome solution (HFS, 0.1% sodium citrate (w/v), 0.1% Triton-X-100 (v/v) in H_2_O) containing 50 µg/mL PI and incubated for 12 h at 4 °C protected from light. Cell populations with a sub-G1 PI signal were considered apoptotic.

For measurement of mitochondria superoxide generation, the MitoSOX Red dye was employed. After stimulation with NCA or DMSO as indicated, cells were resuspended in HHBS containing 5 µM dye and loaded for 30 min at 37 °C protected from light. All flow cytometry experiments were performed using a BD FACS Canto II (BD Biosciences, Franklin Lahes, NJ, USA). Data were evaluated using FlowJow 7.6.

### Immunoblotting

Cells were treated with NCA or DMSO as indicated, washed twice with ice-cold PBS, detached, and centrifuged. The pellet was resuspended in lysis buffer (150 mM NaCl, 50 mM Tris-HCl, 1% NP-40, 0.25% deoxycholate, 0.1% SDS in H_2_O, pH 7.4) supplemented with protease inhibitor cocktail Complete^®^ (Roche, Basel, Switzerland). Subcellular fractionation was performed according to the Abcam (Cambridge, UK) protocol [39]. Protein concentrations were determined by Pierce assay using a BSA standard and adjusted with 5x and 1x sample buffer (312.5 mM Tris pH 6.8, 50% glycerol, 5% SDS, 4% DTT, 0.025% Pyronin Y in H_2_O). Samples were subjected to SDS-PAGE (21 min 100 V, 40 min 200 V), and loading amounts were checked by stain-free technology on a ChemiDoc^TM^ (Bio-Rad Laboratories Inc., Hercules, USA). Proteins were transferred onto 0.45 *µ*m nitrocellulose (NC) membrane by tank-blotting (1:30 h, 100 V, 4 °C). Membranes were washed with TBS-T, blocked with 5% BSA and probed with the following primary antibodies: ATF4 (sc-390063), BiP (BD 610978), Bid (CST 2002), caspase-3 (sc-714), caspase-8 (sc-9746), caspase-9 (sc-9502), cytochrome c CST 4272), eIF2α (sc-133132), p-eIF2α (CST 9721), GAPDH (CST 5174), Hsp60 (sc-1052), pan-cadherin (sc-515872), PARP (CST 9542), Rtn4 (sc-271878). After incubation with the corresponding HRP-linked secondary antibodies, homemade luminol-containing (2.5 mM) ECL solution was used to visualize protein bands using a ChemiDoc^TM^. Images were further processed with Image Lab 6.0. Full-length uncropped original western blots are provided as supplementary data.

### Quantitative real-time PCR

HeLa cells were treated with NCA, DMSO, and thapsigargin at the indicated concentrations for 18 h. Cells were washed twice with ice-cold PBS, and RNA isolation was performed using the RNeasy Mini Kit (74104 Qiagen) according to the manufacturer’s instructions. The obtained mRNA content was determined with a Nanodrop spectrophotometer. 1250 ng mRNA were subjected to reverse transcription using the High-Capacity cDNA Reverse Transcription Kit (Applied Biosystems, Waltham, MA, USA) and rt-qPCR using SYBR^®^ Green Mastermix (Applied Biosystems) (2 µL cDNA (=50 ng), 0.25 µL forward and reverse primer (200 nM), 6.25 µL SYBR^®^ Green Master Mix, 3.75 µL RNAse-free water/well). Experiments were performed with a QuantStudio^TM^ 3 Real-Time PCR System (Applied Biosystems) and data evaluated by the ΔΔCT method as described elsewhere [40]. GAPDH served as housekeeping control. All primers were purchased from Metabion (Planegg, Germany) and primer validation conducted.

### Statistical analyses

All experiments were conducted at least three times (*n* = 3) and presented as mean ± SD if not stated otherwise. Statistical significance between two samples was analyzed by unpaired *t*-test with Welch correction or Mann–Whitney test, between more than two samples by ordinary one-way ANOVA with Dunnet’s posttest, and between two groups and different conditions by ordinary two-way ANOVA with Tukey’s posttest, depending on the respective variances and on the normal distribution of the data. Results were considered statistically significant for *p* < 0.05. Analyses were conducted using GraphPad Prism 10.

## Supplementary information

Text Summary of Supplementary Files

Supplementary information

Supplementary Table 1

Supplementary Table 2

Uncropped Western blots

Supplementary Figure S1

Supplemetray Figures S2-3

Supplementary Figures S4-7

Supplementary Figures S8-10

Supplementary Figures S11-12

Supplementary Figure S13

Supplementary Figures S14-16

## Data Availability

All data generated and analyzed during this study are either included in this article or the supplementary material. Additional information to data might be obtained from the corresponding author upon reasonable request.
