# Supplementary material for: Neocarzilin A induces apoptosis and mitochondrial disturbance by targeting reticulon 4-mediated endoplasmic reticulum stress
Source: Cell Death Discov. 2025 Jun 16;11:278. doi: 10.1038/s41420-025-02560-3 (PMC12170863; doi:10.1038/s41420-025-02560-3)
Supplement: Supplementary file 1 — Text Summary of Supplementary Files [file 41420_2025_2560_MOESM1_ESM.docx]

Text Summary of Supplementary Information Files:

1. Supplementary Information with Supplementary Methods, and Figure Legends to Supplementary Figures (WORD file)
2. Uncropped Western Blots (PDF File)
3. 2 Supplementary Tables: Proteomics data (2 PDF Files)
4. 14 Supplementary Figures (1 PDF File)
