# Supplementary material for: Neocarzilin A induces apoptosis and mitochondrial disturbance by targeting reticulon 4-mediated endoplasmic reticulum stress
Source: Cell Death Discov. 2025 Jun 16;11:278. doi: 10.1038/s41420-025-02560-3 (PMC12170863; doi:10.1038/s41420-025-02560-3)
Supplement: Supplementary file 2 — Supplementary information [file 41420_2025_2560_MOESM2_ESM.docx]

-Supplementary information-

Table of content

[1. Supplementary Methods 3](#_Toc196386165)

[ATP Assay 3](#_Toc196386166)

[Complex I enzymatic assay 3](#_Toc196386167)

[High-resolution respirometry 4](#_Toc196386168)

[Molecular docking 5](#_Toc196386169)

[Oxygen consumption assay 5](#_Toc196386170)

[Transmission electron microscopy 5](#_Toc196386171)

[2. Supplementary figure legends 6](#_Toc196386172)

[3. References 8](#_Toc196386173)

# Supplementary Methods

## ATP Assay

For determination of ATP levels, the CellTiter-Glo^®^ assay kit (Cat # G757, Promega, Walldorf, Germany) was used according to the manufacturer’s instructions. Cells were concomitantly treated with NCA, and the glycolysis inhibitor 2-deoxy-D-glucose (200 mM) for 6 h, ATP synthase inhibitor oligomycin A (5 *µ*g/mL, 1 h) was used as positive control. After adding the CellTiter-Glo reagent and lysing the cells, equal volumes were transferred into an opac-walled plate, and luminescence was measured on an Orion II microplate luminometer (Berthold Detection Systems GmbH, Pforzheim, Germany).

## Complex I enzymatic assay

HeLa cells were treated with 10 *µ*M NCA or DMSO for 6 h before mitochondria were enriched as described before (1). Briefly, cells were detached, pelleted, and resuspended in hypotonic buffer (250 mM sucrose, 10 mM HEPES, 1 mM EDTA in H_2_O pH 7.4). After freezing in liquid nitrogen and thawing at 37 °C, cells were permeabilized with digitonin, and mitochondria-enriched fractions were obtained by centrifugation at 10 000xg. The 10 000xg pellets were resuspended in 25 mM K_2_HPO_4_ (pH 7.4) and subjected to three freeze-thaw cycles in liquid nitrogen. Complex I enzymatic activity was measured by reduction of absorbance of DCIP at 595 nm in the presence of antimycin A. Rotenone was used to assess not complex I-related NADH conversion and subtracted from the rotenone-free proportion. Activity was calculated from the slope of the fitted curve and mitochondrial protein amount (Bradford assay).

$$Enzyme activity\left[ nmol/min/mg \right]=\frac{\Delta A/\min*1000}{\varepsilon\left( DCIP \right)*V\left( sample \right)*c(sample)}$$

$$\varepsilon\left( DCIP \right)=19.1\frac{1}{\frac{mmol}{L}*cm}$$

## High-resolution respirometry

High-resolution respirometry in digitonin-permeabilized cells was performed with an Oxygraph-2k (Oroboros Instruments Corp, Innsbruck, Austria). Cells were seeded the day prior to measurement and on the following day treated with 5 *µ*M NCA or DMSO for 6 h. Then, they were collected, counted, and 1.5x10^6^ cells resuspended in mitochondrial respiration medium MiR05 (0.5 mM EGTA, 3 mM MgCl_2_, 60 mM lactobionic acid, 20 mM taurine, 10 mM KH_2_PO_4_, 20 mM HEPES, 110 mM sucrose, 1 g/L BSA in H_2_O, pH 7.1). For direct treatments, the compound or DMSO was added to the measuring chambers after permeabilization. Cell suspension was added by partial replacement method and protocol SUIT-008_O2_ce-pce_D025 was applied to assess ROUTINE, LEAK, complex I, and combined complex I/II respiration, as well as maximal electron transfer capacity after uncoupling, including the following titration steps: digitonin (1Dig, 10 *µ*g/mL in DMSO), pyruvate + malate (1PM, 5 mM + 2 mM in H_2_O), ADP (2D, 2 mM in H_2_O), cytochrome *c* (2c, 10 *µ*M in H_2_O), glutamate (3G, 10 mM in H_2_O), succinate (4S, 10 mM in H_2_O), CCCP (5U, 0.5 *µ*M in EtOH), rotenone (6Rot, 0.5 mM in EtOH), antimycin A (7Ama, 2.5 *µ*M in EtOH). Data were evaluated using DataLab7 software.

## Molecular docking

Molecular docking of 66 *aa* cytoplasmic/extracellular loop of Nogo (Nogo-66, <https://doi.org/10.2210/pdb2KO2/pdb>, medoid model 3 chosen for docking) and neocarzilin A (structure derived from PubChem, ID 10381557) was done with UCSF Chimera and Autodock Vina plugin. Number of binding modes was set to 6, exhaustiveness to 8 and max. energy difference to 3 kcal/mol. Interactions were made evident by highlighting involved residues of the protein and the “Find Claches and Contacts” function and depicted in yellow lines. Hydrogen bonds were calculated with the “FindHBond” function and depicted in red. Tables of analysis are included in the supplementary information section.

## Oxygen consumption assay

For determination of the oxygen consumption rate (OCR), the MitoXpress Xtra Oxygen Consumption Assay kit (Agilent) was employed according to the manufacturer’s instructions. In brief, HeLa cells were seeded into 96-well plates and medium refreshed directly before measurement. MitoXpress reagent containing an oxygen-quenchable dye was added, and the cells treated with NCA, DMSO, and antimycin A as positive control. The surfaces of the wells were sealed with mineral oil to avoid re-diffusion of oxygen into the medium, and the plate kinetically measured at ex./em. 380 nm/670 nm for 6 h at 37 °C with a Tecan InfiniteF200Pro (Tecan, Männedorf, Switzerland).

## Transmission electron microscopy

For transmission electron microscopy (TEM), HeLa cells were treated with NCA or DMSO as indicated. After 6 h, 3.5x10^5^ cells were transferred to a BEEM^®^ capsule (Electron Microscopy Sciences, Hatfield, PA, USA), pelleted by centrifugation, and fixed with 0.1 M cacodylate buffer containing 2.5% glutaraldehyde. After storage at 4 °C for at least 24 h, samples were post-fixed in 1% osmium tetroxide, dehydrated with acetone, and embedded in epoxide resin.

Ultrathin sections (50-60 nm) were cut using Ultracut E (Reichert und Jung, NY, USA) and stained with UranyLess (Science Services, Germany) and 3% lead citrate (Leica, Germany).

Images were acquired using a Joel 1200 EXII electron microscope (Akishma, Tokyo, Japan) at 60 kV, equipped with a KeenViewII digital camera (Olympus, Hamburg, Germany), and processed with the iTEM software package (anlySISFive, Olympus, Germany).

# Supplementary figure legends

**Figure S1** Effect of NCA on mitochondrial dynamics. Mitochondrial networks were analyzed by immunostaining after treatment with NCA or DMSO for 3 h at the indicated concentrations. Representative images on top panel, nuclei shown in blue, Hsp60 in green (scale bar 25 *µ*m). Brightness was adjusted to improve visibility. Bottom row shows MiNA analysis, purple area represents mitochondrial footprint, green lines mitochondrial length, blue dots connection sites, and yellow dots the end of network structures. Normalized data are presented in bar graphs as mean ± SD, n=3. Statistical significance was analyzed by one-way ANOVA with Dunnett’s posttest compared to mean of DMSO control (ns=not significant).

**Figure S2-3** Effect of NCA on mitochondrial dynamics. **2** Mitochondrial, **3** microtubule networks were stained after treatment with NCA or DMSO for 6 h as indicated. Representative images on top and bottom panel, nuclei shown in blue, **2** MitoTracker™ Deep Red FM, **3** tubulin-β in green (**2** scale bar top 50 *µ*m, bottom 25 *µ*m, **3** scale bar 25 *µ*m). Brightness was adjusted to improve visibility.

**Figure S4-7** Effect of NCA on mitochondrial dynamics and respiration. **4**,**5** Protein levels of **4** mitofusin 1 (Mfn1) and **5** dynamin-1-like protein (Drp1) assessed by immunoblotting. Left panel shows representative blots. **6**,**7** High-resolution respirometry measurements of **6** 6 h or **7** directly NCA or DMSO treated cells following SUIT protocol 008_O2_ce-pce_D025 in an Oroboros O2k instrument. Normalized data are presented in bar graphs as mean ± SD, **4**,**5** n=3, **7** n=4 and **6** n=5 (except CIV respiration, n=2). Statistical significance was analyzed by **6** two-tailed unpaired Student’s t-test or Mann-Whitney test or **4**,**5**,**7** one-way ANOVA with Dunnett’s posttest compared to mean of DMSO control (ns=not significant, *P<0.05, **P<0.01, ***P<0.001).

**Figure S8-10** Assessment of ATP production and effect of NCA on autophagy. **8** CellTiter-Glo assay to determine ATP content of NCA or DMSO stimulated HeLa cells as indicated, without glycolysis inhibition. **9** Cytotoxicity of NCA or DMSO treatment for 6 h and oligomycin for 1 h in combination with 200 mM 2-DG determined by crystal violet staining. **10** Protein levels of Parkin, p62, Atg5, TFAM and PGC-1α assessed by immunoblotting. Left panel shows representative blots. Normalized data are presented in bar graphs as mean ± SD, n=3 (Parkin n=2). Statistical significance was analyzed by one-way ANOVA with Dunnett’s posttest compared to mean of DMSO control (ns=not significant).

**Figure S11-12** Effect of VAT-1 knockout on ROS production and BST-2 on mitochondrial dynamics. **11** Mitochondrial superoxide generation after NCA or DMSO treatment as indicated, measured with the MitoSOX^TM^ dye by flow cytometry in HeLa wt and VAT-1/BST-2 knockout cells. Antimycin A served as positive control. **12** MiNA analysis of mitochondrial networks of siBST-2 knockdown HeLa cells treated with NCA as indicated. **11**,**12** Normalized data are presented in bar graphs as mean ± SD, n=3. Statistical significance was analyzed by **11** two-way ANOVA with Tukey’s posttest or **12** two-tailed unpaired Student’s t-test (ns=not significant, *P<0.05, **P<0.01).

**Figure S13** Effect of VAT-1 knockout on mitochondrial dynamics. Mitochondrial networks in VAT-1 knockout cells vs. HeLa wt and CRISPR control cells were analyzed by immunostainings after treatment with NCA or DMSO for 6 h at the indicated concentrations. Representative images on top and bottom panel, nuclei shown in blue, Hsp60 in green (scale bar 25 *µ*m). Brightness was adjusted to improve visibility. Bottom row shows MiNA analysis, purple area represents mitochondrial footprint, green lines mitochondrial length, blue dots connection sites, and yellow dots the end of network structures. Normalized data are presented in bar graphs as mean ± SD, n=3. Statistical significance was analyzed by one-way ANOVA with Dunnett’s posttest compared to mean of DMSO control (*P<0.05, **P<0.01, ***P<0.001).

**Figure S14-16** Cytoplasmic vacuolization and Rtn4 knockdown. **14**,**15** Cytoplasmic vacuolization observed by phase contrast microscopy of **14** VAT-1 KO HeLa cells treated with NCA or DMSO as indicated and **15** HeLa wt cells treated with NC-4 target probe or DMSO as indicated, n=2 (**14**,**15** scale bar 50 *µ*m). **16** Validation of Rtn4 knockdown efficiency, normalized data are presented in bar graphs as mean ± SD, n=8. Statistical significance was analyzed by Mann-Whitney test (***P<0.001).

# References

1. de Wit LE, Sluiter W. Chapter 9 Reliable assay for measuring complex I activity in human blood lymphocytes and skin fibroblasts. Methods Enzymol. 2009;456:169-81.
