# Supplementary material for: Neocarzilin A induces apoptosis and mitochondrial disturbance by targeting reticulon 4-mediated endoplasmic reticulum stress
Source: Cell Death Discov. 2025 Jun 16;11:278. doi: 10.1038/s41420-025-02560-3 (PMC12170863; doi:10.1038/s41420-025-02560-3)
Supplement: Supplementary file 3 — Supplementary Table 1 [file 41420_2025_2560_MOESM3_ESM.pdf]

Supplementary Table 1

protein name	gene name	enrichment	p-value	sequence coverage [%]
Heme oxygenase 2	HMOX2	5.45	5.44	46.8
Bone marrow stromal antigen 2	BST2	5.23	3.32	18.3
Vesicle amine transport protein 1 homolog	VAT1	3.93	3.85	48.9
Prostaglandin E synthase	hCG_30600	3.42	1.46	5.6
Reticulon-1	RTN1	3.40	4.47	2.4
GTPase-activating protein and VPS9 domain-containing protein 1	GAPVD1	3.14	1.55	6.6
CD63 antigen	CD63	2.95	1.32	13.5
Cytochrome b5 type B	CYB5B	2.71	4.75	45.2
CD44 antigen	CD44	2.69	1.30	20.9
Amino acid transporter	SLC1A1	2.66	2.95	5.5
cDNA FLJ58568	C16orf58	2.54	3.34	9.5
PRA1 family protein	PRAF2	2.43	1.81	20.8
Coactosin-like protein	COTL1	2.36	1.59	21.9
Endonuclease domain-containing 1 protein	ENDOD1	2.27	2.55	14.2
Pirin (Iron-binding nuclear protein)	PIR	2.15	2.08	15.2
cDNA FLJ46477 fis	MCAM	2.11	1.99	15.1
UPF0729 protein C18orf32	C18orf32	2.02	3.12	13.2
(Reticulon-4)	RTN4	1.97	3.59	15.9)

Table S1 ABBP LFQ experiments in HeLa wt cells with 250 nM NC-4 probe (1 h) conducted by Josef B. (TU Munich, Germany) derived from previous study (PXD050453). Proteins matching criteria (p-value < 0.05, log2 fold-change > 2, except Rtn4 in brackets).
