# Supplementary material for: Neocarzilin A induces apoptosis and mitochondrial disturbance by targeting reticulon 4-mediated endoplasmic reticulum stress
Source: Cell Death Discov. 2025 Jun 16;11:278. doi: 10.1038/s41420-025-02560-3 (PMC12170863; doi:10.1038/s41420-025-02560-3)
Supplement: Supplementary file 4 — Supplementary Table 2 [file 41420_2025_2560_MOESM4_ESM.pdf]

Supplementary Table 2

protein name	gene name	enrichment	p-value	sequence coverage [%]
Heme oxygenase 2	HMOX2	6.56	5.52	46.2
Bone marrow stromal antigen 2	BST2	6.27	5.24	18.3
CD63 antigen	CD63	4.40	3.29	13.5
Reticulon-3	RTN3	3.58	2.15	5.4
CD44 antigen	CD44	3.15	1.62	31.7
Cytochrome b5 type B	CYB5B	2.78	4.77	45.2
Serpin peptidase inhibitor	SERPINE2	2.72	1.33	8.7
cDNA FLJ56823		2.36	2.19	9.4
Tubby-related protein 2	TULP2	2.23	1.60	12.6
Ankyrin repeat and KH domain-containing protein 1	ANKHD1	2.13	1.90	2.2
Dolichol-phosphate mannosyltransferase subunit 1	DPM1	2.10	1.47	8.5
Mitochondrial aldehyde dehydrogenase 2 variant	ALDH2	2.10	3.26	36.8
Reticulon-4	RTN4	2.06	2.49	14.3
MICOS complex subunit	APOOL	2.03	1.69	17.5

Table S2 ABBP LFQ experiments in HeLa VAT-1 KO cells with 250 nM NC-4 probe (1 h) conducted by Josef B. (TU Munich, Germany) derived from previous study (PXD050453). Proteins matching criteria (p-value < 0.05, log2 fold-change > 2)
