# Supplementary material for: Neocarzilin A induces apoptosis and mitochondrial disturbance by targeting reticulon 4-mediated endoplasmic reticulum stress
Source: Cell Death Discov. 2025 Jun 16;11:278. doi: 10.1038/s41420-025-02560-3 (PMC12170863; doi:10.1038/s41420-025-02560-3)

-Original Western blots-

Figure 1B

OPA1

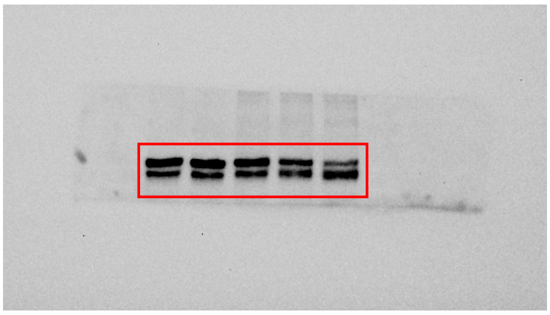

Colorimetric

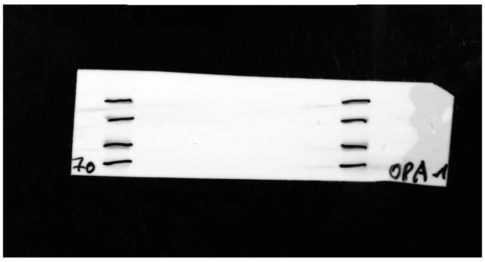

Stain-free loading control

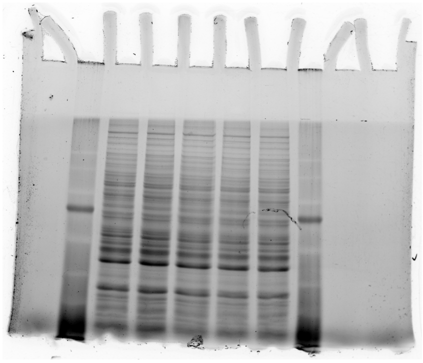

Figure 5A

pan Cadherin

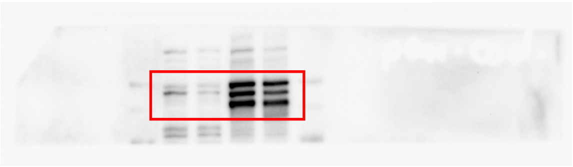

BIP

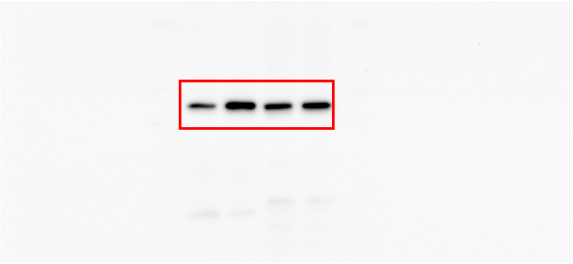

GAPDH

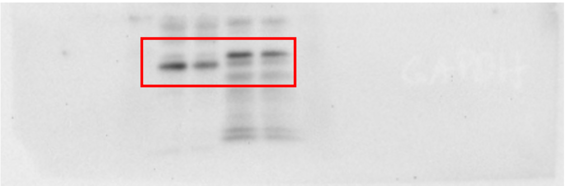

Colorimetric

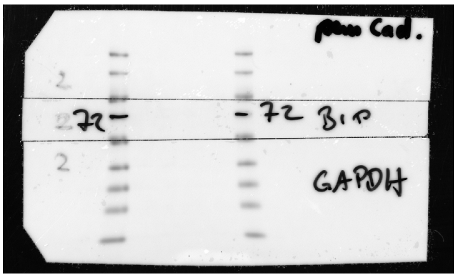

Stain-free loading control

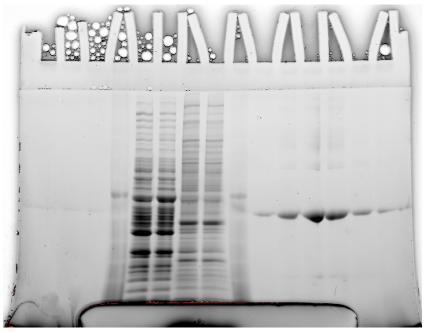

Figure 5B

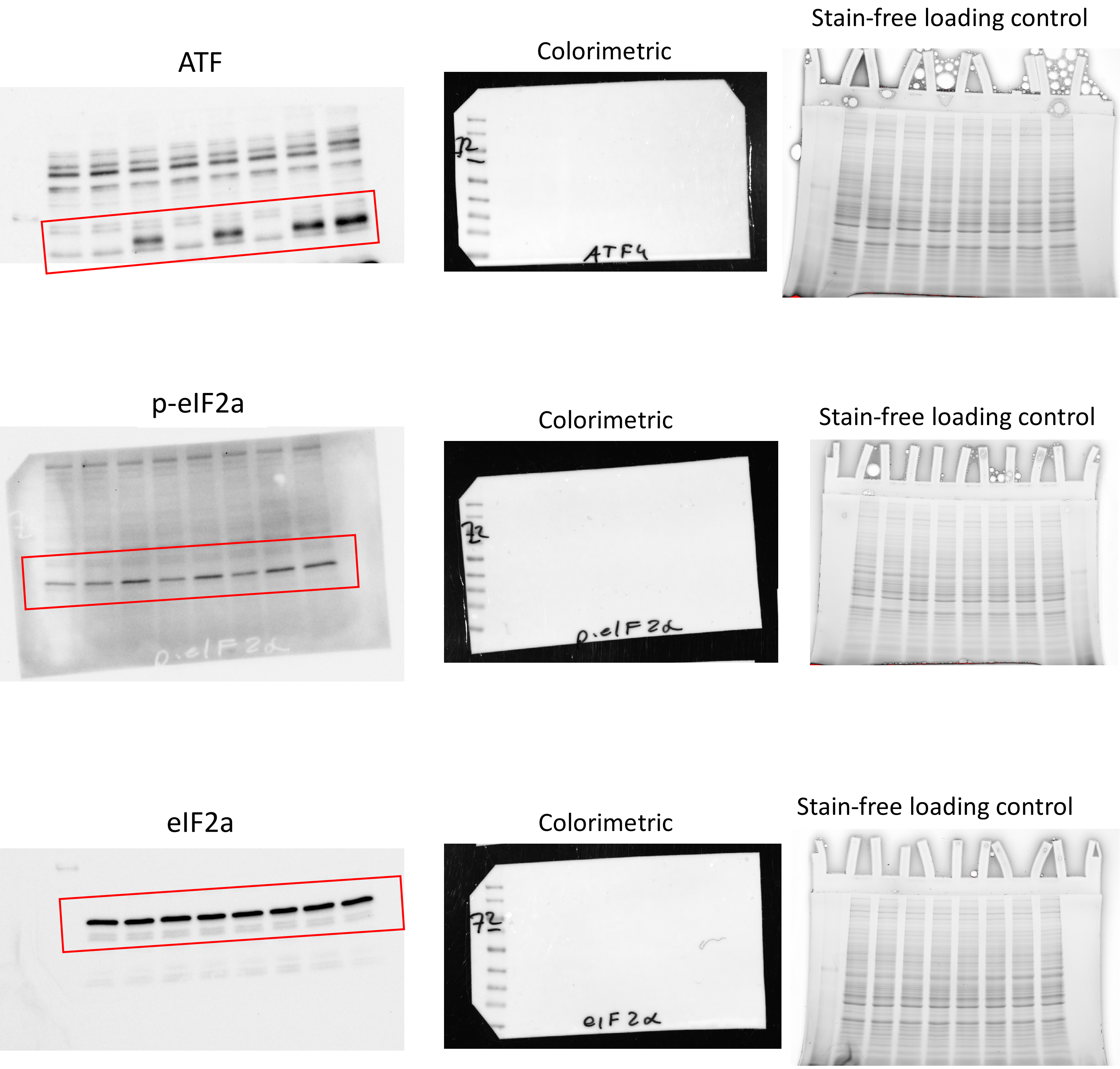
ATF
Colorimetric
Stain-free loading control
ATF4
p-eIF2a
Colorimetric
Stain-free loading control
p.eIF2α
eIF2a
Colorimetric
Stain-free loading control
eIF2α

Figure 6A

Caspase 8

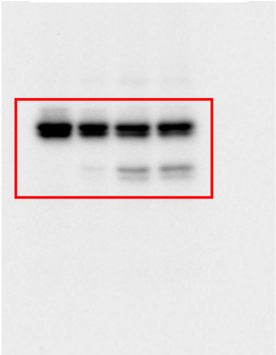

Colorimetric

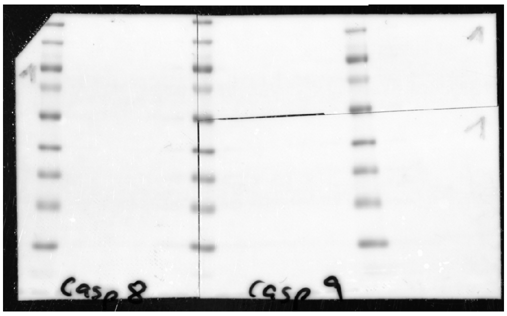

Stain-free loading control

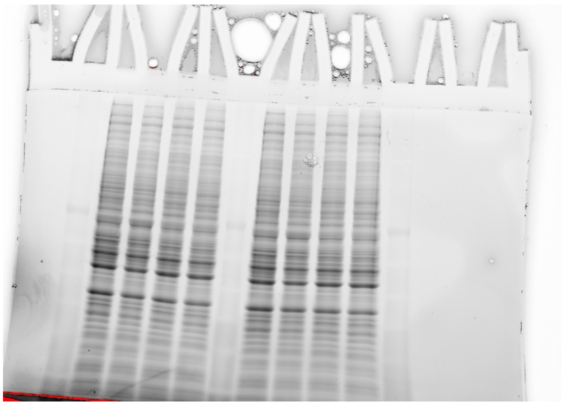

Figure 6B

BID

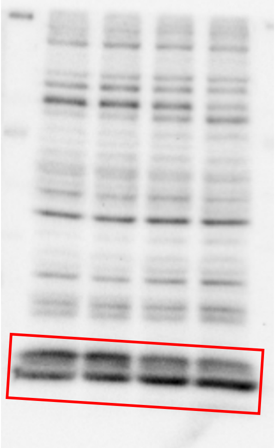

Colorimetric

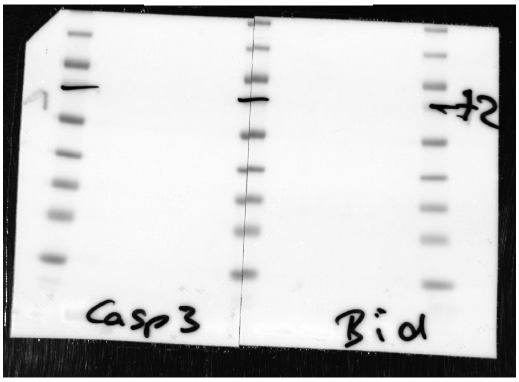

Stain-free loading control

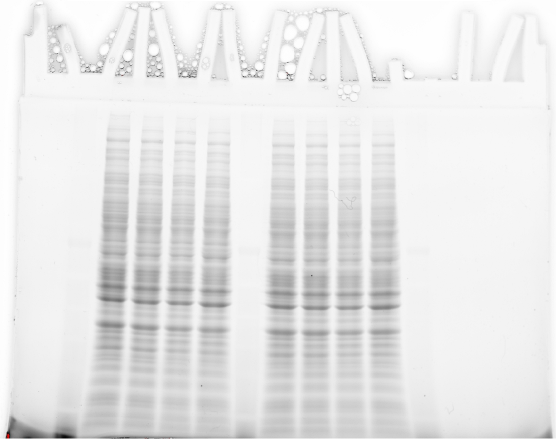

Figure 6C

Caspase 9

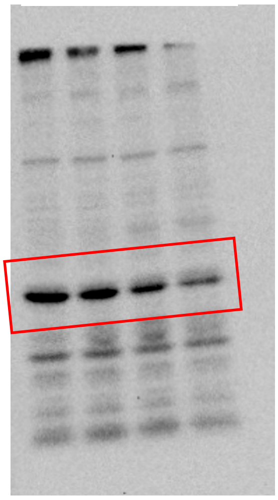

Colorimetric

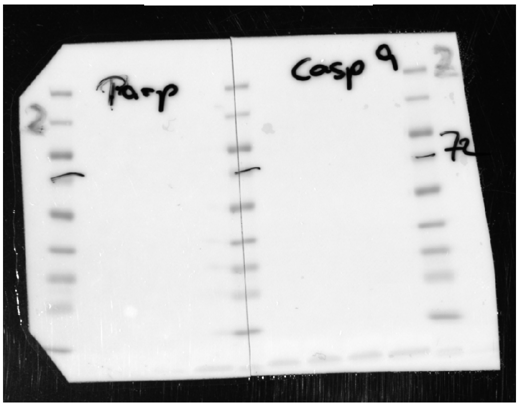

Stain-free loading control

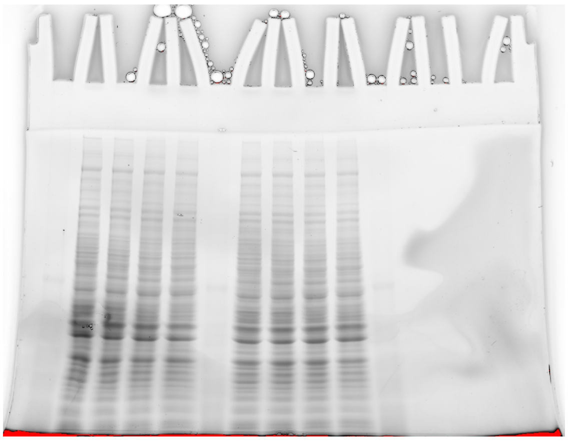

Figure 6D

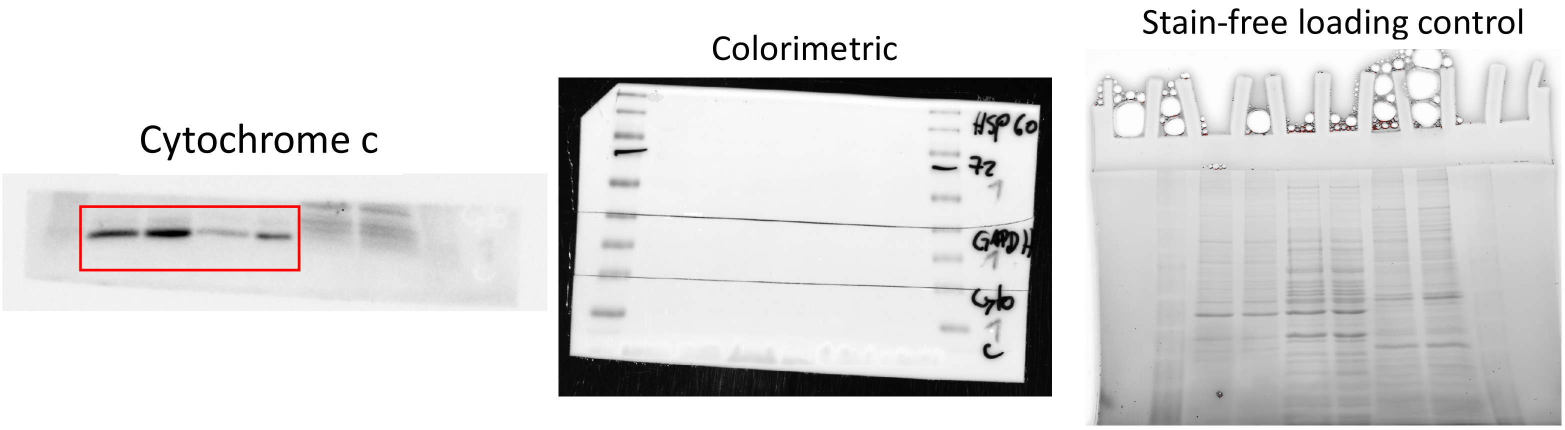

Figure 6E

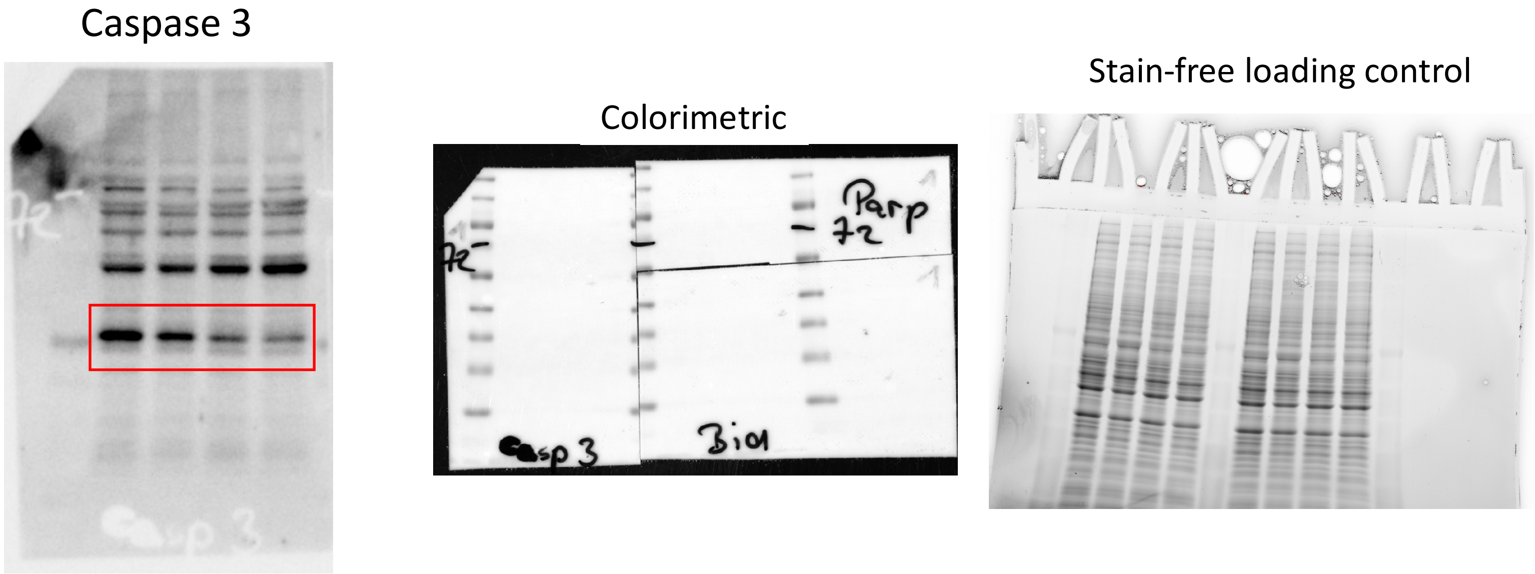

Figure 6F

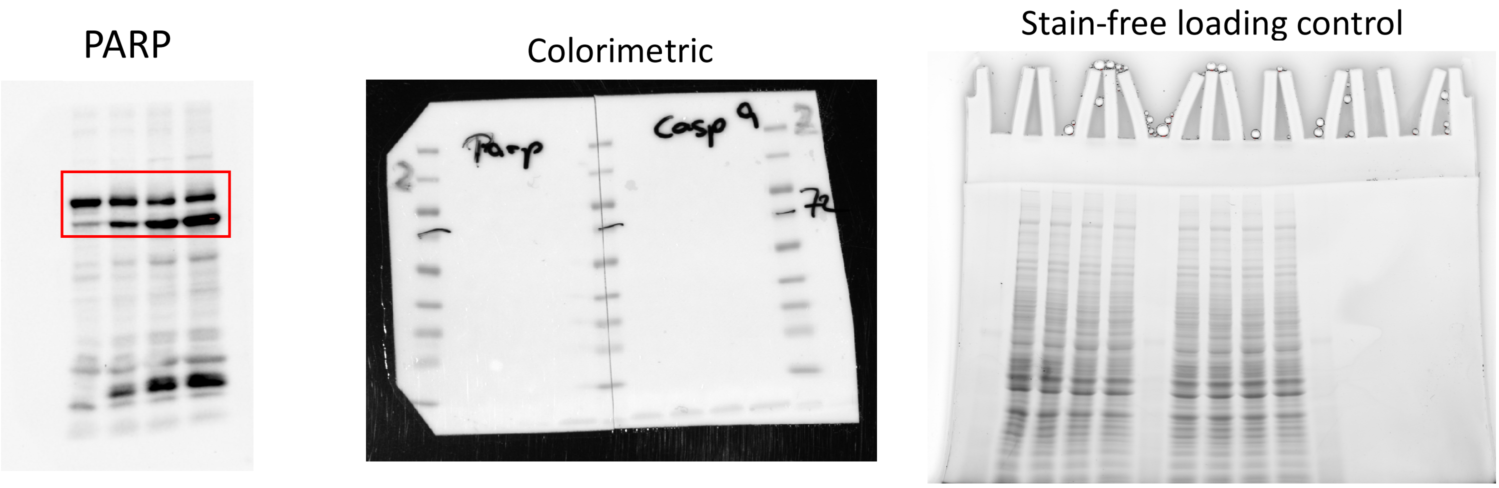

Figure S3

Mitofusin 1

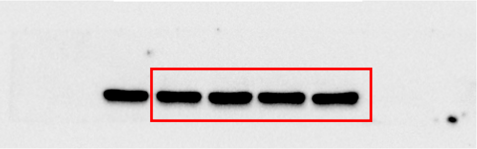

Colorimetric

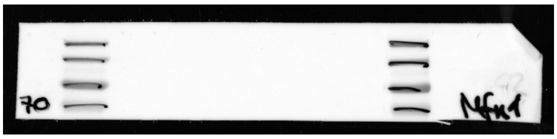

Stain-free loading control

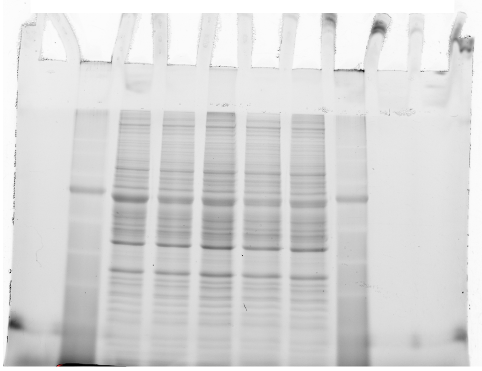

Figure S4

Drp1

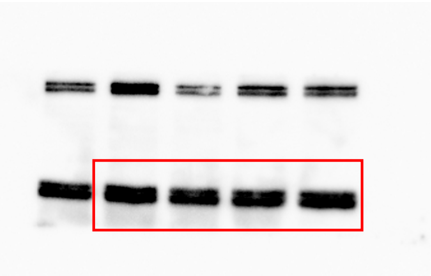

Colorimetric

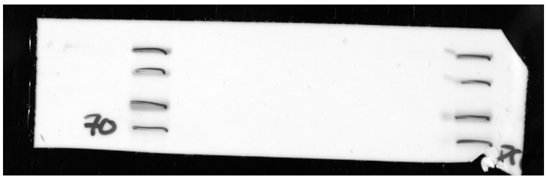

Stain-free loading control

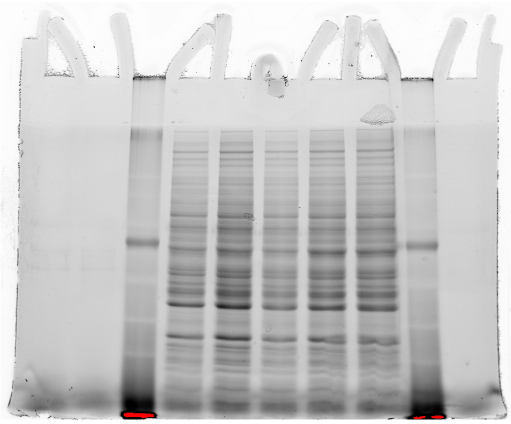

Figure S10

Parkin

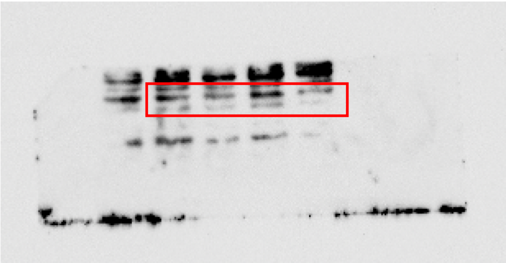

Colorimetric

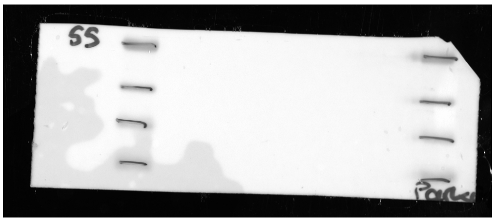

Stain-free loading control

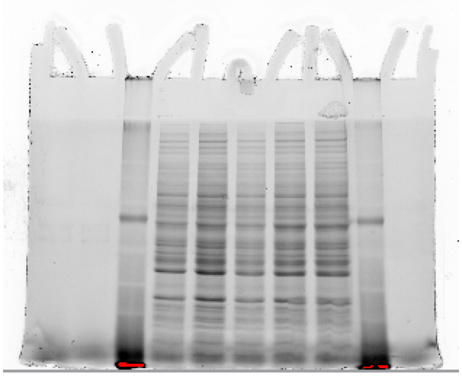

p62

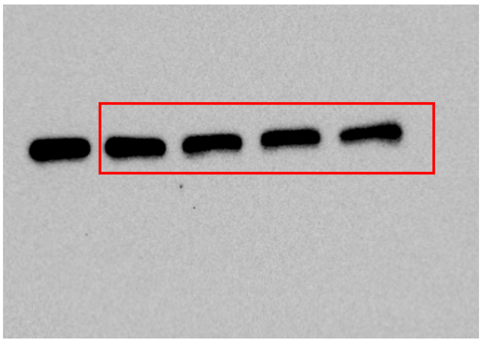

Colorimetric

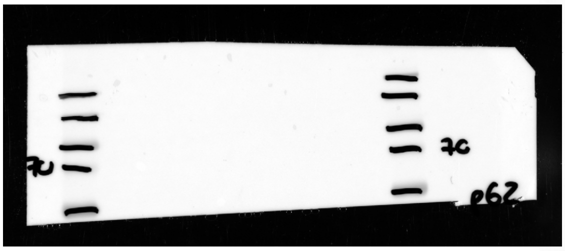

Stain-free loading control

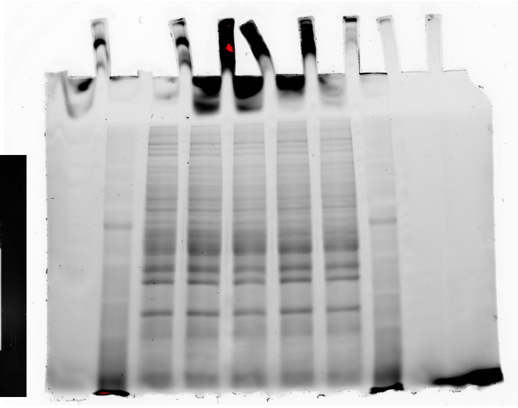

Stain-free loading control

Atg5

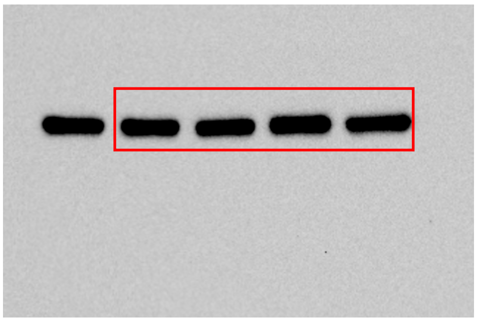

Colorimetric

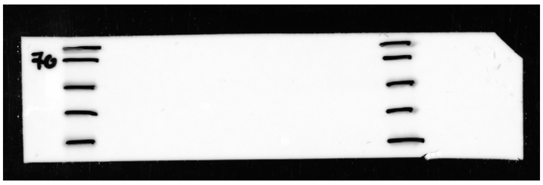

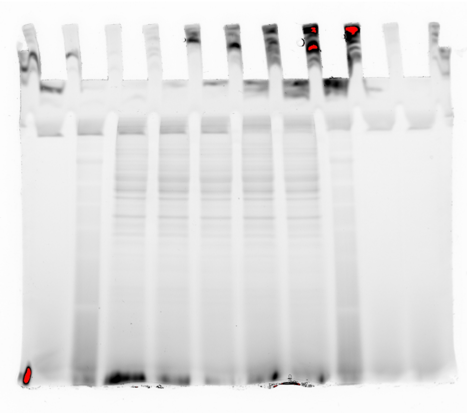

Stain-free loading control

TFAM

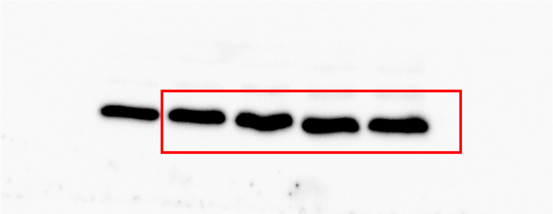

Colorimetric

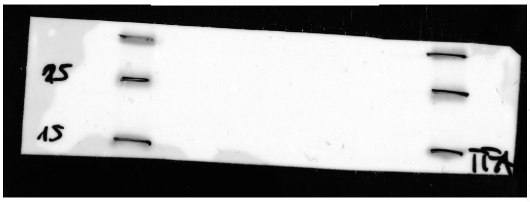

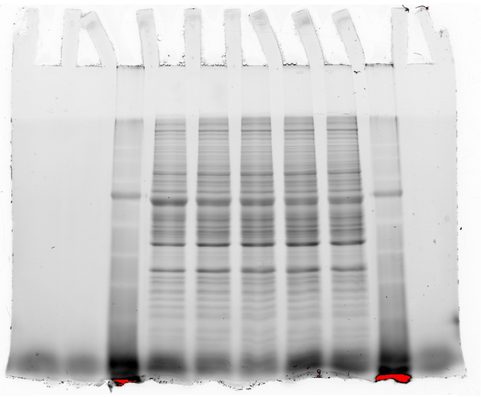

Stain-free loading control

PGC-1α

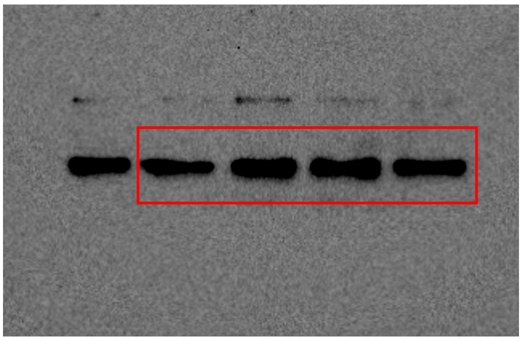

Colorimetric

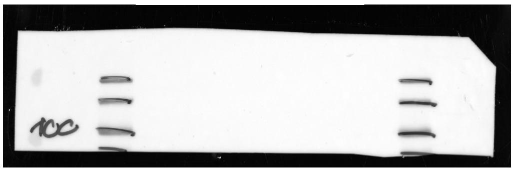

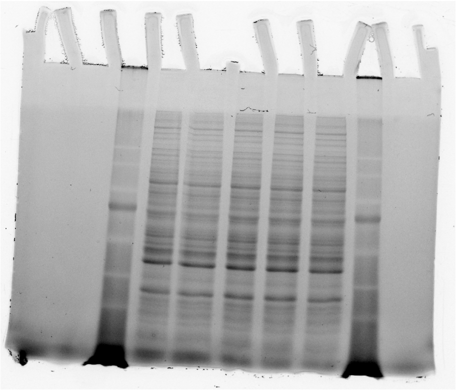

Figure S14

Stain-free loading control

Rtn4

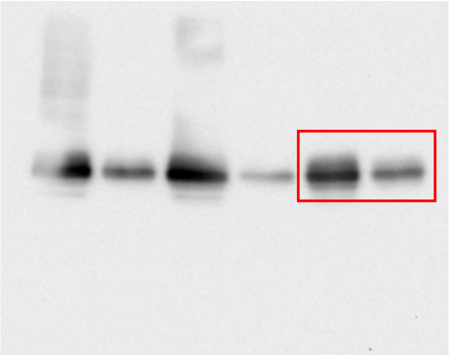

Colorimetric

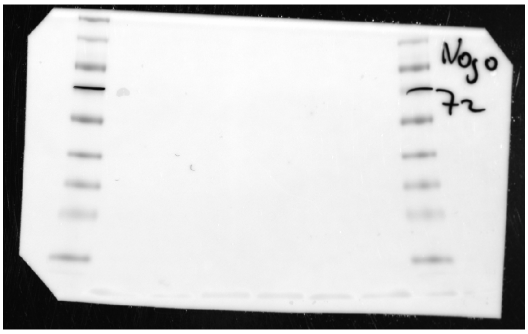

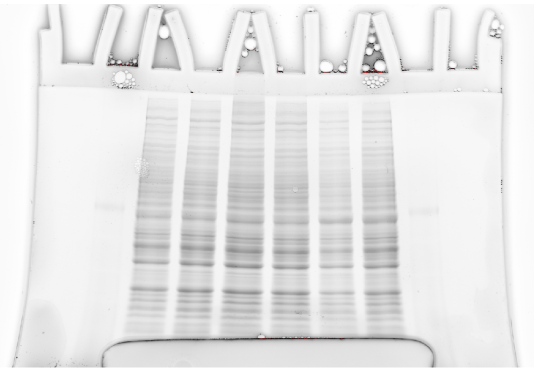

Supplement: Supplementary file 5 — Uncropped Western blots [file 41420_2025_2560_MOESM5_ESM.pdf]
